# Intratumoral antigen signaling traps CD8+ T cells to confine exhaustion to the tumor site

**DOI:** 10.1126/sciimmunol.ade2094

**Published:** 2024-05-24

**Authors:** Munetomo Takahashi, Tsz Y. So, Vitalina Chamberlain-Evans, Robert Hughes, Juan Carlos Yam-Puc, Katarzyna Kania, Michelle Ruhle, Tiffeney Mann, Martijn J. Schuijs, Paul Coupland, Dean Naisbitt, Timotheus Y.F. Halim, Paul A. Lyons, Pietro Lio, Rahul Roychoudhuri, Klaus Okkenhaug, David J. Adams, Ken G.C. Smith, Duncan I. Jodrell, Michael A. Chapman, James E. D. Thaventhiran

**Affiliations:** 1 Medical Research Council Toxicology Unit, University of Cambridge; Gleeson Building, Tennis Court Road, Cambridge, CB2 1QR, UK; 2 Graduate School and Faculty of Medicine, The University of Tokyo, Tokyo, 113-0033, Japan; 3 University of Cambridge, CRUK Cambridge Institute; Cambridge, CB2 0RE, UK; 4 Altos Labs Cambridge Institute, Cambridge CB21 6GP, UK; 5 Department of Pharmacology and Therapeutics, University of Liverpool; Sherrington Building, Ashton Street, Liverpool, L69 3G, UK; 6 Cambridge Institute of Therapeutic Immunology and Infectious Disease, University of Cambridge; Jeffrey Cheah Biomedical Centre, Cambridge Biomedical Campus, Cambridge, UK; 7 Department of Medicine, University of Cambridge, School of Clinical Medicine; Cambridge Biomedical Campus, Cambridge, UK; 8 Department of Computer Science and Technology, University of Cambridge; Cambridge, CB3 0FD, UK; 9 Department of Pathology, University of Cambridge; Cambridge, UK; 10 Experimental Cancer Genetics, Wellcome Sanger Institute; Hinxton, Cambridge, CB10 1SA; 11 The Walter and Eliza Hall Institute of Medical Research, Parkville, VIC 3052, Australia; 12 The University of Melbourne, Parkville, VIC 3052, Australia; 13 Department of Oncology, University of Cambridge, School of Clinical Medicine; Box 197, Cambridge Biomedical Campus, Cambridge, CB2 0XZ, UK; 14 Department of Hematology, University of Cambridge, Cambridge, CB2 0RE, UK

## Abstract

Immunotherapy advances have been hindered by difficulties in tracking the behaviors of lymphocytes following antigen signaling. Here we assessed the behavior of T cells active within tumors through the development of the antigen receptor signaling reporter (AgRSR) mouse, fate mapping lymphocytes responding to antigen at specific times and locations. Contrary to reports describing the ready egress of T cells out of the tumor, we find that intratumoral antigen signaling traps CD8+ T cells in the tumor. These clonal populations expand and become increasingly exhausted over time. By contrast, antigen signaled regulatory T cell (Treg) clonal populations readily recirculate out of the tumor. Consequently, intratumoral antigen signaling acts as a gatekeeper to compartmentalize CD8+ T cell responses, even within the same clonotype, thus enabling exhausted T cells to remain confined to a specific tumor tissue site.

## Introduction

CD8+ T cell exhaustion is an epigenetically propagated (1, 2), temporally increasing (3) permanent state of hypofunction that prevents damaging immune responses (4, 5). Studies using elegant parabiosis experiments (6, 7) and Kaede mice (8, 9) have shown exhausted CD8+ T cells resident in tumor tissue. These findings corroborate reports of exhausted T cells expressing CD103 (a key protein in T cell residency) (10, 11), and reports of tumor-reactive cells being largely confined to the tumor (12, 13). Recent studies have also observed the egress of CD8+ T cells, including antigen-specific T cells, out of the tumor (8, 9). Consequently, it remains unclear what precise factors regulate CD8+ T cell tumor residency and egress.

Upon receipt of antigen signals, naïve T cells undergo clonal expansion to form clonotypes. The subsequent antigen signals they encounter remain pivotal in modulating their responses. Since antigen signaling is contingent on the timing and location of interaction, its effect varies for individual cells. Accordingly, antigen signaling could play a role in segregating the functional response of T cells, including regulating the residency or egress behaviors of cells from the same clonotype (7, 9). Current tools to track lymphocyte responses *in vivo*, primarily using TCR transgenic lymphocytes (14–16) and tetramers (17, 18), cannot distinguish if, where, and when a T cell has received antigen signals. As a result, an alternative approach that tracks the effect of antigen signaling would help in validating its role in regulating CD8+ T cell tumor residency and egress.

## Results

### Developing the antigen receptor signaling reporter (AgRSR) mouse to fate-map antigen-signaled T cells

**Fig. 1:**
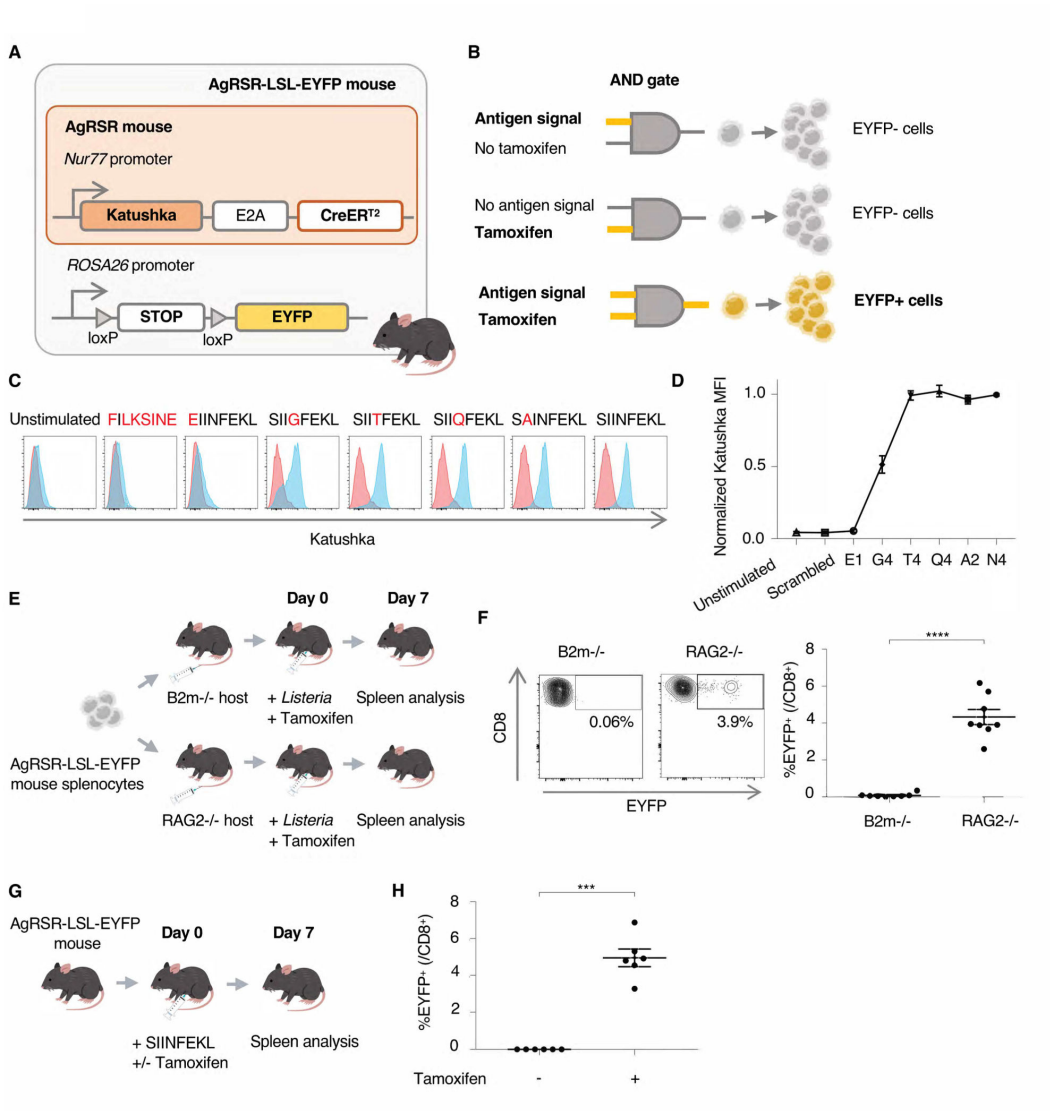
The AgRSR mouse fate-maps antigen-signaled CD8+ T cells. (**A**) Transgenes in the antigen receptor signaling reporter (AgRSR) and AgRSR-LSL-EYFP mouse. (**B**) Lymphocytes receiving antigen and tamoxifen signaling, and their progenies are marked EYFP+. (**C-D**) Range of Katushka expression of OT-I x AgRSR-LSL-EYFP and WT CD8+ T cells following stimulation with SIINFEKL variant peptides, by flow cytometry histograms (**C**) and normalized fluorescence (**D**). Data are representative of two independent experiments. (**E**) AgRSR-LSL-EYFP splenocytes were adoptively transferred to B2m^-/-^ and RAG2^-/-^ strains, challenged with *Listeria* and tamoxifen treated. (**F**) Representative flow cytometry plots of splenocytes at day 7 post infection (Left), and summary plot of all mice (Right). Data are pooled from two independent experiments (n=7 per condition). (**G**) AgRSR-LSL-EYFP mice were immunized with SIINFEKL with or without tamoxifen. (**H**) Summary plot of all mice. Data are pooled from three independent experiments (n=6 per condition). Dots represent mice (**F, H**). Mean ± SEM as shown (**D, F, H**). Statistical testing via unpaired two-tailed students t-test (****, p <0.0001; ***, p <0.001).

We created the antigen receptor signaling reporter (AgRSR) mouse to enable a repertoire-wide assessment of clones that are contemporaneously TCR-signaled, including from tissues in which their cells are rare. In the AgRSR mouse the *Nur77* promoter, which is only active in T lymphocytes upon T cell receptor (TCR) ligation (19, 20), drives equimolar expression of a red fluorescent protein (Katushka) and Cre-ERT2 recombinase (Fig. 1A). Crossing AgRSR mice to Rosa-Lox-Stop-Lox (LSL)-EYFP strains generates AgRSR-LSL-EYFP mice in which the transgenic system acts as a molecular AND-gate that permanently marks the T cells and their progeny that have received coincident TCR and tamoxifen signals with EYFP (Fig. 1B). We first crossed the AgRSR strain to the OVA specific OT-I TCR transgenic strain, for which variant peptide ligands of the TCR have been characterized (21). Naïve CD8+ T cells from these animals were stimulated with OVA peptide variants *in vitro*. Consistent with results from a previous *Nur77*-*GFP* (19) strain, the level of Katushka induced by each ligand directly correlated with its stimulatory activity (Fig. 1C-D), indicating Katushka expression is dependent on the strength of TCR signaling. Next, to validate the TCR-pMHC dependence of EYFP expression in vivo, we transferred splenocytes from AgRSR animals into B2m knockout (KO) and RAG2 KO host mice (Fig. 1E and Fig. S10A). In these CD8+ T cell deficient strains, the MHC-I TCR-ligand is absent in the B2m but present in the RAG2 KO animals. Recipient mice infected with *Listeria* and treated with tamoxifen showed EYFP+ fluorescence in CD8+ T cells from the RAG2 KO but not the B2m KO recipients (Fig. 1F). This confirmed that EYFP expression was dependent on physiological TCR signaling caused by peptide-MHC-I ligation rather than other inflammatory signals present in *Listeria*-infected mice. Equivalent assays to test specificity of labelling to MHC Class II antigen receptor signals were performed for CD4+ T cells (Fig. S1A-B). Lastly, we validated EYFP expression dependence on tamoxifen signals. EYFP expression was only detected in CD8+ T cells of tamoxifen-treated mice with no detectable fluorescence in the absence of tamoxifen (Fig. 1G-H). By temporally staggering injection of TCR-activated CD8+ T cells into tamoxifen-treated mice, we assessed the duration of tamoxifen signal inducing EYFP expression *in vivo*. In line with previous studies (22), the majority of CD8+ T cells were EYFP labelled within a 48-hour window after tamoxifen injection, with no EYFP labelling occurring 72 hours after tamoxifen injection (Fig. S2A-B). Together, these results demonstrate the ability of the AgRSR mouse to fate-map T cells on coincident TCR and tamoxifen signals in vivo.

### CD8+ T cell exhaustion is confined to the tumor site

**Fig. 2:**
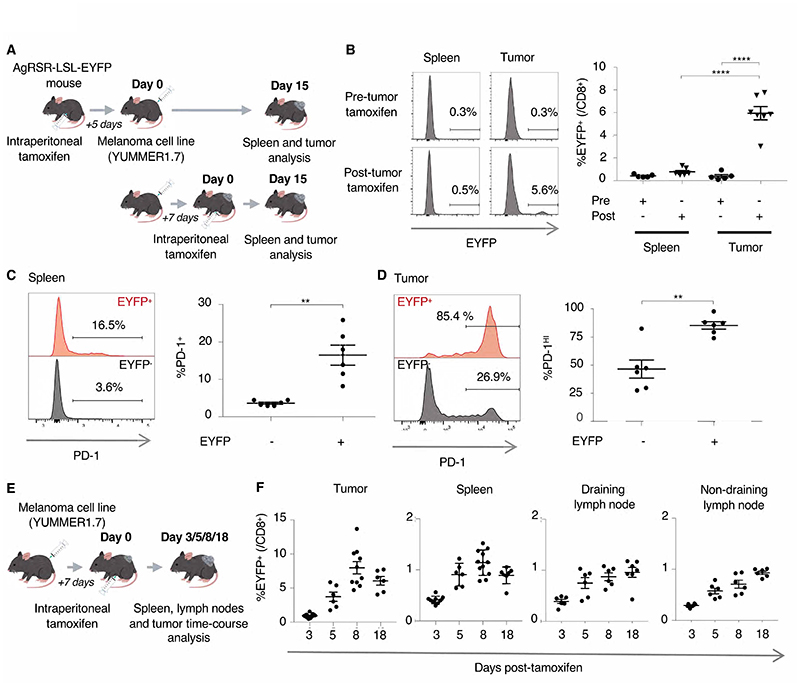
The AgRSR mouse tracks expansion of antigen-signaled CD8+ T cells in the tumor immune response. (**A**) AgRSR-LSL-EYFP mice were tamoxifen treated pre- (5 days before) or post- (7 days after) subcutaneous injection of YUMMER1.7 melanoma cells, and CD8+ T cells were assessed 15 days later. (**B**) Representative flow cytometry histograms (Left) and summary plot of all experiments (Right). Data are pooled from two independent experiments (n≥5 per condition). (**C-D**) Representative flow cytometry histograms (Left) and summary plot (Right) of PD-1 expression in CD8+ T cells from the spleen (**C**) and the tumor (**D**) of CD8+ T cells 8 days after tamoxifen labelling. Data are pooled from two independent experiments (n=6). (**E**) AgRSR-LSL-EYFP mice were implanted with YUMMER1.7 melanoma cells, treated with tamoxifen 7 days later, and CD8+ T cells from the spleen, tumor, draining lymph node and non-draining lymph node were sampled at indicated days. (**F**) EYFP+ percentages of CD8+ T cells in the indicated tissues. Data are pooled from four independent experiments (n≥5 per condition). Dots represent mice (**B, C, D, F**). Mean ± SEM as shown (**B, C, D, F**). Statistical testing via paired two-tailed students t-test and ordinary one-way ANOVA (****, p <0.0001; **, p <0.01).

The YUMMER1.7 melanoma model provides a neoantigen-rich persistent immunological challenge that has been used for the characterization of CD8+ T cell responses during immunotherapy (23–25). We utilized the model to investigate the effect of antigen signaling on T cell responses. When implanted, tumors grew consistently in tamoxifen treated AgRSR mice (Fig. S3A). Only AgRSR mice receiving tamoxifen after, and not before tumors became palpable, showed elevated CD8+ T cell tumor EYFP+ frequency (Fig. 2A-B and Fig. S10B). In tumor-bearing mice, splenic EYFP+ CD8+ T cells expressed PD-1 and tumor EYFP+ CD8+ T cells were PD-1^Hi^ (Fig. 2C-D), indicating substantial EYFP enrichment of T cells responding to tumor antigens in both populations (26, 27). We quantified the longitudinal changes in EYFP+ frequency of CD8+ T cells in the secondary lymphoid system and tumor (Fig. 2E). EYFP+ frequencies expanded in all compartments, with particularly high EYFP+ frequency expansion observed in the tumor (Fig. 2F). This heightened expansion could be due to direct labelling of intratumorally antigen-signaled cells, in which a subset of cells within a clonotype that receive TCR signals in the tumor is labelled EYFP+. To assess whether tamoxifen was indeed labelling subsets of clonotypes or whole clonotypes, we performed bulk TCR-seq of CD8+EYFP+ and CD8+EYFP- cells 8 days after tamoxifen administration (Fig. S3B). Since naïve T cells carry a unique TCR sequence inherited by progenies during clonal expansion, checking for TCR overlap between the EYFP+ and EYFP- compartments would indicate the extent of clonotype labelling. Most cells from the EYFP+ compartment shared TCR sequences with cells from the EYFP- compartment across all tissues (Fig. S3C), indicating EYFP labelling capturing subsets of clonotypes. Taken together, these results demonstrate the AgRSR system’s ability to characterize the consequences of antigen signaling in the tumor immune response.

**Fig. 3:**
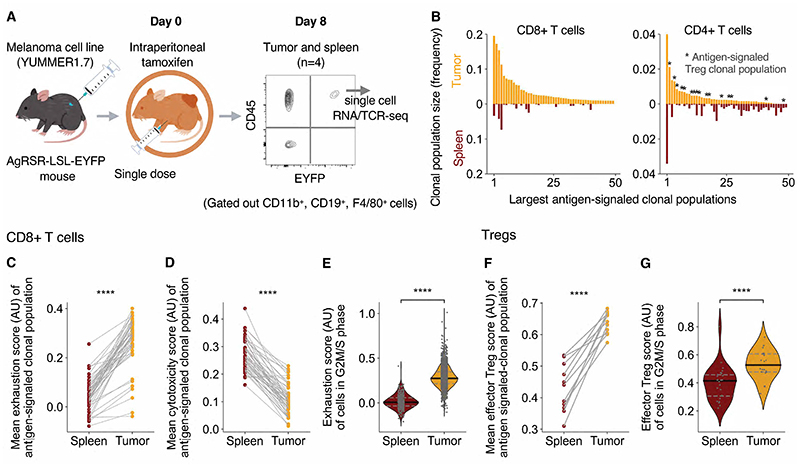
Antigen-signaled CD8+ T and Treg cells partition their differentiation state by tissue site. (**A**) EYFP+ T cells were sorted from the tumor and spleen of AgRSR-LSL-EYFP mice 8 days after intraperitoneal tamoxifen injection and subject to single-cell RNA and VDJ analysis (n=4). (**B**) Largest antigen-signaled T cell clonal populations ranked by size, displaying clonal population size (frequency) within the tumor or spleen. (**C-G**) Antigen-signaled CD8+ T cell (**C-E**) and Treg (in **F-G**) clonal populations with at least two cell members in both the tumor and spleen assessed for mean exhaustion (**C**), cytotoxicity (**D**) and effector Treg gene set expression score (**F**) in each tissue. Scores from the same antigen-signaled clonal population are linked by a line. (**E, G**) Violin plot comparing the gene set scores of individual cells in G2M/ S phase across tissues. Dots represent cells (**E, G**) and antigen-signaled clonal populations (**C, D, F**). Statistical testing via paired two-tailed students t-test and Kruskal-Wallis test (****, p <0.0001). (AU) arbitrary units.

To investigate the differentiation of antigen-signaled T cells at both the effector site and the circulating immune system, we analyzed sorted EYFP+ T cells from the tumor and spleen of 4 mice by paired scRNA-Seq and TCR sequencing, 8 days after tamoxifen injection (Fig. 3A). The TCR sequence provided a genetic barcode, which, in conjunction with EYFP labelling, enabled identification of T cell clonal expansions after antigen signaling (“antigen-signaled clonal populations”). In the dataset, most EYFP+ cells from the spleen were non-expanded, whereas most EYFP+ cells from the tumor were members of expanded antigen-signaled clonal populations (Fig. S4A). In the largest (> 15 cells) antigen-signaled clonal populations (Fig. 3B), CD8+ and CD4+ T cell clonal populations could be readily distinguished by their *Cd8a* and *Cd4* expression (Fig. S4B-C). Antigen-signaled CD4+ T cell clonal populations were further filtered based on the proportion of cells expressing *Foxp3* (Fig. S4D). We focused on a subset of CD4+ “Treg” clonal populations that consistently expressed *Foxp3. Foxp3* expressing cells from these clonal populations highly expressed genes associated with the Treg state (including *Foxp3, Helios* and *Nrp1*) (Fig. S4E).

We first compared the differentiation states of antigen-signaled CD8+ T cell clonal populations across the spleen and the tumor. Individual CD8+ T cells were scored for exhaustion and cytotoxicity signatures based on their upregulation of exhaustion and cytotoxicity associated genes (28) against a control group of genes (29). For antigen-signaled CD8+ T cell clonal populations distributed across the spleen and tumor, cells within the tumor had reduced cytotoxicity and elevated exhaustion scores relative to their counterparts within the lymphoid tissue (Fig. 3C-D). Correspondingly, cells defined by their gene expression to be in a cycling phase had higher exhaustion scores in the tumor than in the lymphoid tissue (Fig. 3E). These observations were consistent in individual mice (Fig. S5A) and reproduced when we utilized exhaustion gene sets obtained in different immune settings (30–32) (Fig. S5B). In a human lung cancer dataset (33), tumor infiltrating CD8+ T cell clonal populations that did not have cells detectable in the blood had higher exhaustion scores than counterpart populations that had cells detectable in the blood (Fig. S5C). In line with observations in mice, clonal populations that were detectable in the blood had higher exhaustion scores in the tumor than the blood (Fig. S5D). Together, these data are consistent with a containment of CD8+ T cell exhaustion to the tumor site. We next compared the effector Treg state of Tregs clonal populations across the spleen and the tumor by using an effector Treg differentiation signature (34). In parallel to antigen-signaled CD8+ T cell clonal populations, antigen-signaled Treg clonal populations distributed across the spleen and the tumor, on average, had higher effector Treg scores in the tumor than in the spleen (Fig. 3F). Cells in a cycling phase in the tumor had higher effector Treg scores than equivalent cells in the spleen (Fig. 3G).

### Intratumoral antigen signals trap CD8+ T cells in the tumor

**Fig. 4:**
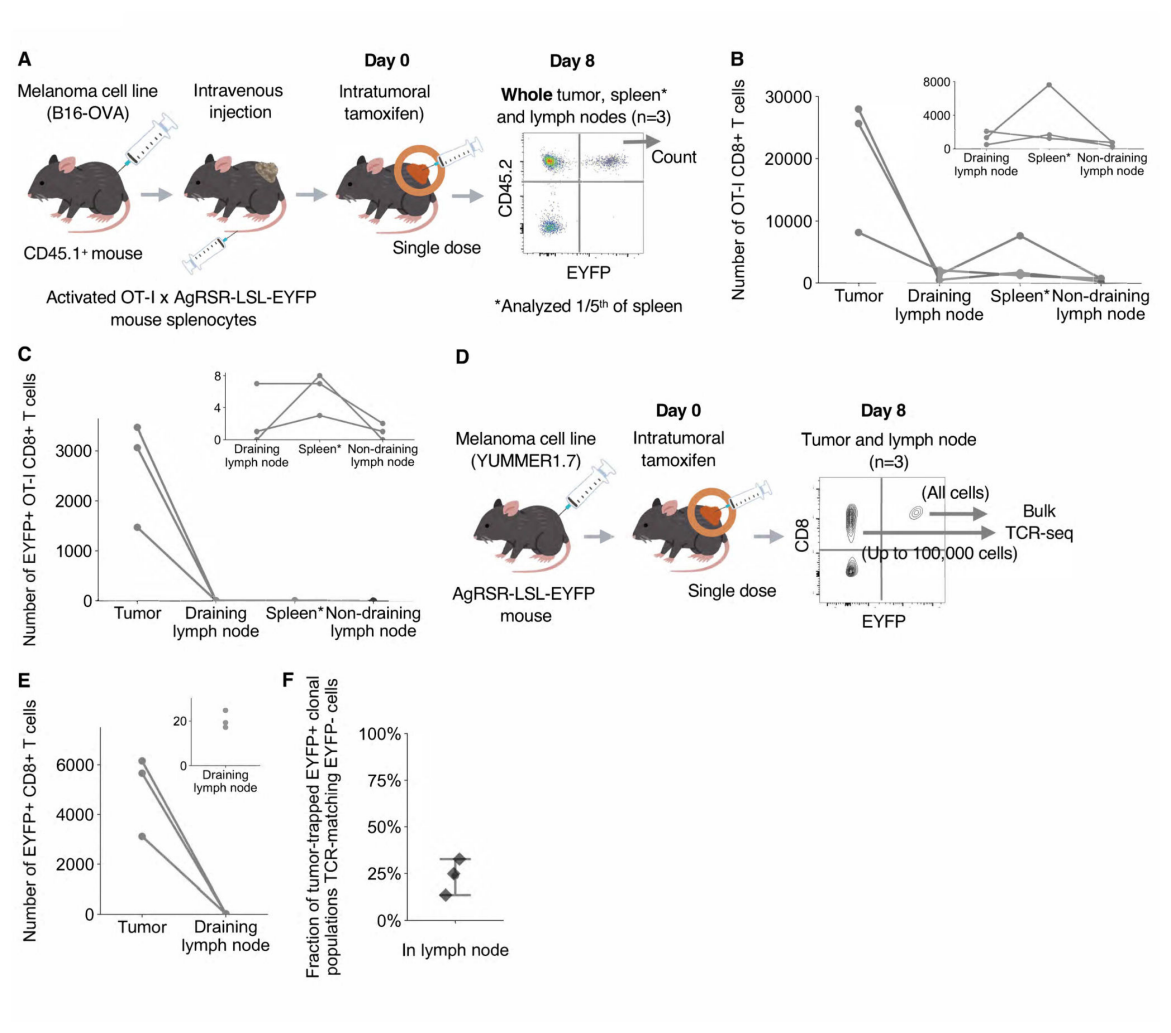
Intratumoral antigen signaling traps CD8+ T cells. (**A**) OT-I CD8+ T cells from the whole tumor, draining and non-draining lymph nodes, and 1/5^th^ of the spleen were analyzed 8 days after intratumoral tamoxifen administration of B16-OVA bearing congenic marker mice that had been injected with activated OT-I x AgRSR-LSL-EYFP mice 4 days earlier (n=3). (**B-C**) Distribution of OT-I CD8+ T cells (**B**) and EYFP+ OT-I CD8+ T cells (**C**) across different tissues with enlarged view of lymphoid tissues (Top right of respective figures). Asterisk denotes 1/5^th^ of the spleen was sampled. (**D**) EYFP+ and EYFP- CD8+ T cells were sorted from the tumor and draining lymph node of AgRSR-LSL-EYFP mice 8 days after intratumoral tamoxifen injection and subject to bulk TCR-seq analysis (n=3). The top 200 largest EYFP+ CD8+ T cell clonal populations in the tumor of each mouse were analyzed. (**E**) Distribution of cells across the tumor and draining lymph node, with enlarged view of the draining lymph node (Top right). (**F**) Fraction of EYFP+ clonal populations detected exclusively in the tumor that had TCR overlap with EYFP -cells in the draining lymph node. Dots represent mice (**B, C, E, F**).

We queried how CD8+ T cells confine their exhausted state to the tumor site. Researchers using the Kaede system, which tracks the migration of all photoconverted CD8+ T cells, have demonstrated egress of tumor T cells (including activated, antigen-specific CD8+ T cells) to the lymphoid system (8, 9, 35), consistent with views that effector CD8+ T cells re-enter the recirculating immune system (36, 37). To investigate the effect of intratumoral antigen signaling on CD8+ T cell responses, we implanted congenic marker mice with B16-OVA melanoma tumors and injected activated TCR transgenic OT-I CD8+ AgRSR-LSL-EYFP cells specific for the SIINFEKL peptide in OVA (Fig. 4A). Mice were then treated intratumorally with tamoxifen, which labels antigen-signaled T cells in the tumor (Fig. S6A-C). After 8 days, despite OT-I CD8+ T cells being detected in both the tumor and the lymphoid tissues (Fig. 4B), EYFP+ cells were detected almost exclusively in the tumor (Fig. 4C). The OVA/OT-I system models an exceptionally high, single antigen-TCR interaction. We therefore utilized the AgRSR system to investigate the effect of intratumoral signaling in a system for which a normal T cell repertoire can be activated by diverse neoantigens. YUMMER1.7 implanted AgRSR-LSL-EYFP mice were intratumorally injected with tamoxifen, and all CD8+EYFP+, and a proportion of the CD8+EYFP- cells from the tumor and lymph node were processed through bulk TCR-seq 8 days after intratumoral tamoxifen administration (Fig. 4D). Again, EYFP+ cells were detected almost exclusively in the tumor (Fig. 4E). Despite limited sampling of the EYFP-cells, a large fraction of clonal populations had overlapping TCRs with EYFP-cells in the lymph node (Fig. 4F). Taken together, these data demonstrate that intratumorally antigen-signaled CD8+ T cell clonal populations become trapped in the tumor, even while cells from the same clonotype reside outside the tumor.

**Fig. 5:**
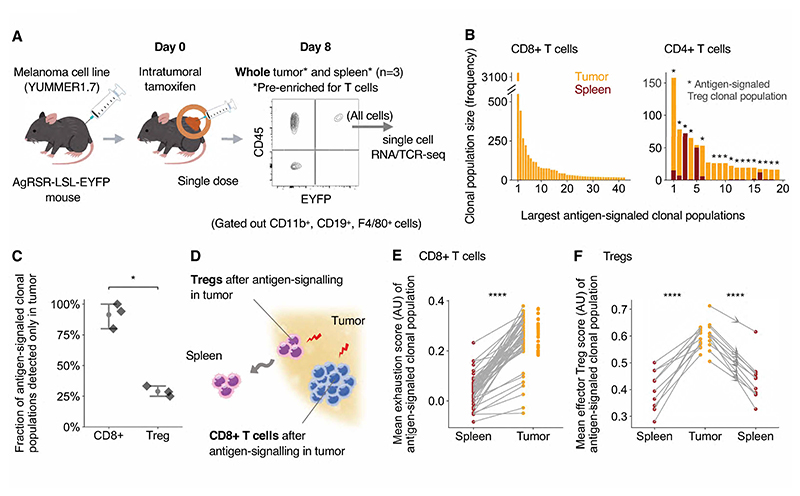
Intratumoral antigen-signals trap and exhaust CD8+ T cells but not Tregs. (**A**) EYFP+ T cells were sorted from the whole pre-enriched tumor and spleen of AgRSR-LSL-EYFP mice 8 days after intratumoral tamoxifen injection and subject to single-cell RNA and VDJ analysis (n=3). (**B**) Largest antigen-signaled T cell clonal populations ranked by size and colored by tissue origin. (**C**) Fraction of antigen-signaled CD8+ T cell and Treg clonal populations for which no cells with the same TCR were identified in EYFP+ cells of the spleen. (**D**) Effect of intratumoral antigen signaling in CD8+ T cell and Treg clonal populations. (**E-F**) The average exhaustion and effector Treg score of antigen-signaled CD8+ T cell (**E**) and Treg (**F**) clonal populations obtained from intraperitoneal fate-mapping (Left section – as in Fig. 3C, 3F) and intratumoral fate-mapping (Right section and labelled with arrows for Treg clonal populations). Dots represent antigen-signaled clonal populations (**E, F**) and mice (**C**). Statistical testing via paired two-tailed students t-test and Kruskal-Wallis test (****, p <0.0001; *, p<0.05). (AU) arbitrary units.

To investigate the transcriptomic characteristics of intratumorally antigen-signaled T cells, we analyzed sorted EYFP+ T cells from the tumor and spleen in 3 mice, 8 days after intratumoral injection, by paired single cell RNA and TCR sequencing (Fig. 5A). Both antigen-signaled CD8+ and CD4+ T cell clonal populations were substantially expanded in the tumor (Fig. 5B). In this experiment, EYFP+ cells were sorted from the whole spleen and tumor after magnetic bead enrichment for T cells. Consistent with our previous findings, cells of almost all antigen-signaled CD8+ T cell clonal populations were trapped and confined to the tumor (Fig. 5C-D). Tissue-trapping of activated T cells was not an artefact of the system as Treg clones were readily detected in the spleen and tumor compartments (Fig. 5C-D). We assessed if tissue-trapped clonal populations were labelled following intraperitoneal tamoxifen. Half of the identified CD8+ T cell clones were confined to the tumor, whereas more antigen-signaled Treg clonal populations were distributed across both compartments (Fig. S7A). Both fate-mapping strategies revealed antigen-signaled CD8+ T cell clonal populations to be proliferating (Fig. S7B and S7D), to contain a subset of *Tcf7* expressing stem-cell like cells with a capacity for self-renewal (38) (Fig. S7C and S7E) and to be exhausted in the tumor (Fig. 5E). We investigated how Treg states change after intratumoral antigen signaling. The effector Treg score decreased as cells left the tumor (Fig. 5F). Taken together, these data show that intratumoral antigen signaling generates tissue-trapped, exhausted, expanded, proliferating and self-renewing CD8+ T cell clonal populations in tumors.

### Clustering time-stamped pseudotime trajectories with TrajClust reveals determinants of CD8+ T cell clonal population differentiation

**Fig. 6:**
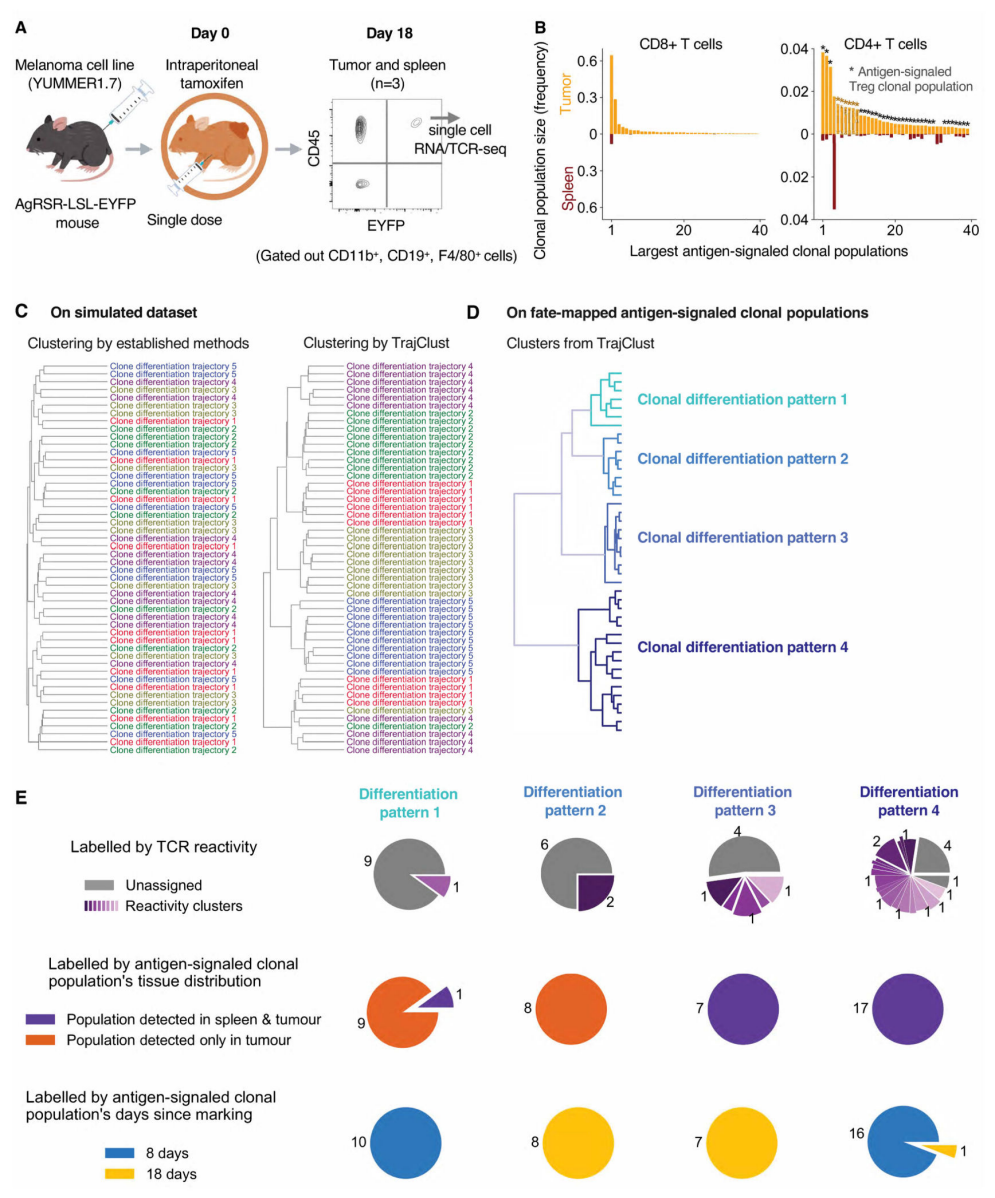
Time elapsed since antigen signaling impacts the differentiation state of CD8+ T cells. (**A**) EYFP+ T cells were sorted from the tumor and spleen of AgRSR-LSL-EYFP mice 18 days after intraperitoneal tamoxifen injection and subject to single-cell RNA and VDJ analysis (n=3). (**B**) Largest antigen-signaled T cell clonal populations ranked by size, displaying clonal population size (frequency) within the tumor or spleen. (**C**) TrajClust, a computational algorithm to cluster clonal differentiation patterns, was applied to simulated datasets containing clonal populations with five different differentiation patterns. Results from an unsupervised clustering of these clonal populations by established methods (Left) and TrajClust (Right) (**D**) Unsupervised clustering of the largest antigen-signaled CD8+ T cell clonal populations found 8 days (Fig. 3A) or 18 days (Fig. 6A) after intraperitoneal tamoxifen injection by TrajClust. Each cluster is denoted clonal differentiation pattern 1-4. (**E**) Pie chart showing the properties of clonal populations from each clonal differentiation pattern. Clonal populations are labelled by their TCR reactivity groups identified by GLIPH2 analysis (Top), their tissue distribution (Middle) and their time elapsed since antigen signaling (Bottom).

Finally, we investigated the response of antigen-signaled T cell clonal populations over time. We undertook paired single cell RNA and TCR sequencing analysis of EYFP+ T cells from the tumor and spleen at day 18 after intraperitoneal tamoxifen treatment in 3 mice (Fig. 6A). For all T cells, most cells in the spleen remained nonexpanded, whereas most cells in the tumor were comprised of expanded clonal populations (Fig. S9A). Again, we focused on the largest, expanded antigen-signaled clonal populations (Fig. 6B). Combining this dataset with our previous intraperitoneal dataset produced an atlas containing antigen-signaled CD8+ T cell clonal populations with diverse reactivities. First, we sought to categorize these clonal populations to define major classes of clone differentiation. Established methods to compare clonal populations (39–41) base comparisons on differentiation state distributions which lose key information about the similarities between clonal populations. We therefore developed TrajClust, an algorithm to cluster clonal populations based on transcriptome-wide differentiation trajectory similarities (Supplementary Methods). Simulated datasets of distinct clone differentiation patterns demonstrated that TrajClust could successfully discover clusters of clonal differentiation patterns that an established clone clustering method using UMAP based similarities could not (Fig. 6C, Fig. S8A-G). When TrajClust was applied to our atlas, four major clonal differentiation patterns were identified (Fig. 6D, Fig. S8H), characterized by groups of differentially expressed genes in the tumor (Data File S1). We queried whether reactivity to a specific antigen could account for these patterns using GLIPH2 (grouping of lymphocyte interactions by paratope hotspots)^26^, an algorithm that identifies reactivity clusters within TCR sequences from multiple donors, but we found no single reactivity driving this clustering. The major clusters corresponded to the time since antigen signaling and the tissue-confinement of the antigen-signaled clonal populations (Fig. 6E). These results therefore demonstrate that irrespective of reactivity, the differentiation of antigen-signaled clonal populations is consistent and is chiefly determined by the duration of their persistence.

### Time elapsed since antigen signaling impacts the tissue distribution of differentiated T cell clonal populations

**Fig. 7:**
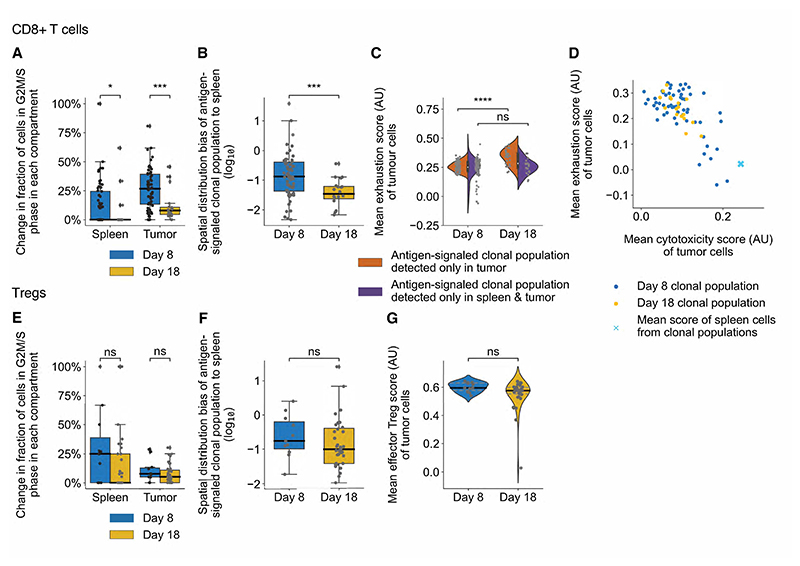
Spatial distribution changes of antigen-signaled T cell clonal populations. (**A-G**) Tracking changes in antigen-signaled T cell clonal populations over days 8 and 18. (**A, E**) Changes in the fraction of cells in G2M/ S phase across tissues for antigen-signaled CD8+ T cell (**A**) and Treg (**E**) populations. (**B, F**) Changes in the spatial distribution bias of antigen-signaled CD8+ T cell (**B**) and Treg (**F**) clonal populations. The spatial distribution bias was calculated by dividing the clonal population size (frequency) in the spleen by that of the tumor. (**C, G**) Changes in the mean exhaustion and Treg effector gene set score of tumor cells from antigen-signaled CD8+ T cell (**C**) and Treg (**G**) clonal populations. (**D**) Mean exhaustion and cytotoxic scores of antigen-signaled CD8+ T cell clonal populations, restricted to analysis of their cells from the tumor (Small dots) or the secondary lymphoid tissue (Large cross – mean of all clonal populations). Individual clonal populations are colored by time elapsed since antigen signaling. Dots represent antigen-signaled clonal populations (**A-G**). Statistical testing via paired two-tailed students t-test and Kruskal-Wallis test (****, p <0.0001; ***, p <0.001; *, p<0.05; ns, p>0.05). (AU) arbitrary units.

These results raised the question of how defined functional states were affected by the duration of population persistence. Again, we filtered the CD4+ T cell clonal populations by *Foxp3* expression. Both antigen-signaled CD8+ T cell and Treg clonal populations continued to express genes indicative of tissue-dependent differentiation, as observed at day 8 (Fig. S9B-E). We analyzed changes in the antigen-signaled CD8+ T cell and Treg clonal populations between days 8 and 18. In antigen-signaled CD8+ T cell clonal populations, the fraction of cycling cells decreased, with almost no antigen-signaled clonal populations containing cycling cells in the spleen by day 18 (Fig. 7A). A lower fraction of cells from antigen-signaled CD8+ T cell clonal populations were also found in the spleen (Fig. 7B). We assessed changes in exhaustion and found this to significantly increase in antigen-signaled clonal populations found exclusively in the tumor (Fig. 7C and S9D). There was no significant change in the average exhaustion of clonal populations containing cells in both the lymphoid system and tumor. The overall exhaustion of some clonal populations was low at day 8, but no clones with low exhaustion were present at day 18. Since the level of cytotoxicity in cycling secondary lymphoid system cells remained constant (Fig. S9F), we hypothesized that this would mean CD8+ T cell clonal populations lose their influx of cytotoxic cells over time and become exhausted. Although some clonal populations within the tumor appeared cytotoxic and functional on day 8, these did not exist at the latter timepoint on day 18 (Fig. 7D). In antigen-signaled Treg clonal populations, neither the fraction of cycling cells, the spatial distribution of the antigen-signaled clonal population, or the effector Treg score changed significantly between days 8 and 18 (Fig. 7E-G).

## Discussion

In this study, we developed and validated a fate-mapping mouse to track lymphocytes based on antigen signaling. Our system enables the marking of lymphocytes that respond to antigen signals at different times and locations. We successfully validated the system’s specificity for exclusively marking lymphocytes (and their progenies) that have received antigen signaling *in vivo*. We note that intratumoral tamoxifen injection may leak as a barely detectable number of EYFP+ events were recorded from T cells taken from extratumoral sites (Fig. S6A-C). Any leakage would work against our findings by marking T cells as extratumorally activated, thus increasing the likelihood of detecting EYFP+ cells in the lymphoid tissues, but we highlight the limitation of this injection method for potential users. Alongside capacity for fate-mapping CD8+ T cells, this system tracks (self-) antigen-stimulated conventional and Treg CD4+ T cells. These cell types maintain tissue homeostasis in response to pathogen, autoimmune, and sterile inflammatory challenges and the application of this system could provide insights into myriad aspects of infection, autoimmunity, and cancer.

Using the AgRSR system, we report how intratumoral antigen signals act as a gatekeeper to compartmentalize CD8+ T cell responses. The antigen receptor of the CD8+ T cell, the TCR, evaluates only antigenic-structure to determine clone selection (42). It cannot, by itself, evaluate the pathogenicity of the antigenic source nor the load and distribution of the antigen. Sustained work over the last three decades has demonstrated how the former constraint is overcome by innate immune recognition signals, but no mechanism has been proposed to address the latter constraint, despite millennia of coevolution with pathogens necessitating a need to balance pathogen control with destructive immune responses. Our work suggests a mechanism by which tissue-specific antigen signaling confines CD8+ T cell activity to a particular tissue niche. CD8+ T cells are primed by an ‘initial’ hit, whereas a ‘second’ hit at the effector site both engages and commits a subset of these cells to the tissue niche. Through this ‘two-hit’ mechanism, CD8+ T cells that have engaged with antigen and become exhausted cannot compromise systemic protection. In the context of chronic pathogens, immunity could therefore separate its responses between tissues with insurmountably high antigenic load and tissues with surmountable antigenic load, enabling pathogen control to be balanced with organism survival.

## Materials and Methods

### Study design

The objective of this study was to investigate the effect of antigen signaling on T cell responses. We developed a reporter mouse and applied it to a murine tumor model to compare responses of T cells receiving antigen-signals systemically and intratumorally. All mouse experiments were performed with random assignment of mice without investigator blinding, with at least 2 biological replicates per experimental group. All data describe biological replicates unless otherwise stated.

### BAC Clone Modification and Purification

A BAC clone containing the *Nr4a1* gene (BACPAC resources) was modified by introducing Katushka E2A linked CreERT2-SV40 polyadenylation signal into the start ATG of the *Nur77* gene by homologous recombination (43). BAC DNA was purified from 200ml bacterial cultures by alkaline lysis (Qiagen buffers), and circular DNA was separated by CsCl ultracentrifugation. Briefly, 4.04g of CsCl were added to 4ml resuspended DNA and CsCl dissolved at 40°C. 25μl 10mg/ml EtBr and 75μl water were added. Samples were spun in a bench tube centrifuge at 3000 rpm for 15 minutes to remove remaining proteins. The DNA CsCl solution was spun at 70,000g for 6 hours. EtBr was removed by n-butanol extraction and the DNA precipitated. Successful recombination was confirmed by PCR. The DNA was spot dialyzed on Millipore VSWPO2500 filters into polyamine buffer (10mM Tris-Cl, pH 7.5, 0.1mM EDTA, 100mM NaCl, 30μM spermine, and 70μM spermidine).

### Experimental Mice

AgRSR mice were generated via pronuclear injection of the modified BAC DNA into 0.5 d fertilized ova of C57BL/6 donors. Founder lines were assessed for transgene expression and the line with the highest expression was crossed with the ROSA26-LSL-EYFP mice (gift from Prof. Doug Winton, CRUK-CI, Cambridge). The AgRSR, AgRSR-LSL-EYFP, OT-I x AgRSR-LSL-EYFP, B2m^-/-^ (Jax, 002087) and RAG2^-/-^ (Jax, 008449) mice were maintained in CRUK Cambridge Institute Biological Resources Unit and University of Cambridge Central Biomedical Service under specific-pathogen-free conditions. All animal experiments were conducted when mice were between 8-12 weeks of age and were conducted in accordance with Home Office guidelines.

### Listeria, Vaccine and Tumor Challenges

Mice were infected with 1500 cfu of *Listeria monocytogenes* in experiments indicated in the text. For immunization, mice were intraperitoneally injected with 50**μ**g SIINFEKL peptide, 10**μ**g anti-CD40 (Bio X cell) and 10**μ**g Poly:IC (InvivoGen). For the generation of murine tumors, cells of the cultured YUMMER1.7 cell line (23) (a gift from M. Bosenberg) or the B16-OVA cell line (44) (a gift from R. Roychoudhuri) were detached with 0.5% trypsin-EDTA (Gibco) for 3 minutes, quenched with complete media, and washed in PBS three times. Single-cell suspensions of 1 million cells (YUMMER1.7) or 200,000 cells (B16-OVA) were subcutaneously injected into the right flank of each mouse. In vivo tumor volumes were monitored by (Width x Depth x Length)/2 using a caliper.

### Tamoxifen Administration

Tamoxifen (20mg/ml) was prepared in EtOH (5% v/v) and sunflower oil (95% v/v) before dissolvement in a 37°C water bath under sonication (35kHz) for 15 minutes. Tamoxifen (2mg) was administered by intraperitoneal injection 24 hours after the *Listeria* challenges, at 0 and 12 hours after the vaccine challenges, 5 days before tumor implantation in the tamoxifen pre-tumor challenge or at day 7 after subcutaneous tumor implantation in all other tumor experiments. For fate-mapping antigen signaling within the tumors, 10**μ**l of 4-hydroxytamoxifen (4OHT) at (39mg/ml) was injected into tumors at day 10 after subcutaneous tumor injection.

### Generation of Single-Cell Suspensions from Tissues

Lymph nodes and spleens were homogenized in PBS/ 0.1% FCS/ 2mM EDTA and filtered (70**μ**m or 100**μ**m filters). RBC lysis buffer (NH_4_Cl/ NaHCO_3_/ EDTA) was used to lyse splenic erythrocytes. Tumors were cut into pieces by a scissor and digested using the Miltenyi Tumor Dissociation Kit, according to the manufacturer instructions, before filtering (100μm then 70μm) to generate a single cell suspension.

### Culturing T cells

Purified T cells from the spleen, and where applicable, cells from the tumor, draining and non-draining lymph nodes, were cultured in complete RPMI (10% FBS, 55 mM 2-Mercapthoethanol). Media was supplemented with IL-2 (20ng/ml), IL-7 (2ng/ml), the SIINFEKL peptide and its variants at (5ug/ml), and incubated at 37°C and 5% CO2 for 24 hours to check for Katushka expression or for 72 hours to check for EYFP expression.

### Flow Cytometry

Surface antibody staining was performed by incubating cells with antibodies against CD3 (17A2: BV421 and PE-Cy7 – BioLegend), CD3e (45-2C11: FITC – eBioscience, 145-2C11: APC/Fire750 – BioLegend), CD4 (RM4-5: BV650 and BV785 – BioLegend, GK1.5: BV605 – BioLegend), Cd8a (53-6.7: APC and BV650 – BioLegend), CD11b (M1/70: BV510, PE/Cy7 and APC/Fire750 – BioLegend), CD19 (6D5: BV510 and PE/Cy7 – BioLegend), CD44 (IM7: PE and APC/Fire750 – BioLegend), CD45 (30-F11: APC – eBioscience, 30-F11: BV785 – BioLegend), CD45.1 (A20: PE – BioLegend), CD45.2 (104: BV786 – BD Horizons), CD62L (MEL-14: BV421 and APC – BioLegend), CD69 (H1.2F3: APC/Fire750 – BioLegend), F4/80 (BM8: PE/Cy7 – BioLegend), CD279 (29F.1A12: BV421 – BioLegend), NK1.1 (S17016D : PE/Cy7 – BioLegend) and Ter119 (TER119: PE/Cy7 – Biolegend). Staining was conducted in PBS containing 0.1% FCS and 2mM EDTA for 30 minutes at 4°C to cells that had been pre-incubated with TruStain FcX (anti-mouse CD16/32). This was preceded by LIVE/DEAD Fixable Aqua or Blue (InVitrogen) staining in PBS (1:100) for 10 minutes at room temperature for dead cell exclusion. Samples were fixed by incubating in buffer containing 1% formaldehyde/0.02% sodium azide/glucose/PBS for 10 minutes at room temperature. Data was acquired on a LSR Fortessa (BD Bioscience) flow cytometer and further analyzed by Flowjo v10 (Treestar). Example gating strategies as shown (Fig. S10).

### Preparation of cells for Bulk TCR-seq and Single Cell RNA/TCR-seq

Cell suspensions from the tumor, spleen, draining, and non-draining lymph nodes were generated independently. Samples were stained as for FACS analysis for 30 minutes on ice. Live immune cells were sorted using a FACS Aria instrument (BD Bioscience) with a 100μm nozzle. For bulk TCR-seq, T cells were pre-enriched from spleen single cell suspensions by magnetic bead purification using Miltenyi beads. The whole tissue was sorted to capture all EYFP+, and 100,000 EYFP- CD8+ T cells from each sample. Sorted cells were collected in Buffer RLT Plus buffer (Qiagen) with 20mM DTT. Lysate was vortexed for 1 min and stored at −80 °C for subsequent analysis. For single cell RNA/TCR-seq, cells were sorted into PBS containing 10%FCS and 2mM EDTA in cold and spun down at 300g 7minutes 4°C before resuspension in PBS to achieve a final concentration of 10-20,000 cells/32**μ**l. Totalseq C Hashtag antibodies were added to individual tissues at 0.1mg/ml prior to scRNA/TCR-seq to barcode spleen samples to enable pooled library preparation; cells were then washed twice and sorted. In the intratumoral injection experiment, T cells were pre-enriched from tumor and spleen single cell suspensions by magnetic bead purification, and the whole sample was sorted.

### scRNA/TCR-seq analysis

Single-cell RNA-seq libraries were prepared using Chromium Single Cell and V(D)J Enrichment Kits following the Single-Cell V(D)J Reagent Kits User Guide (Manual Part CG000086 Rev H, I, J, K, L, M; 10X Genomics). The data from the experiment using intraperitoneal tamoxifen were generated using chemistry (5’ v 1) before the introduction of dual-indexing strategy; the data for the intratumoral 4OHT experiment was generated using chemistry (5’ v 2). Sorted samples were resuspended in PBS-0.04% BSA and loaded into Chromium microfluidic chips to generate single-cell gel-bead emulsions using the Chromium controller (10X Genomics). RNA from the barcoded cells for each sample was reverse transcribed in a C1000 Touch Thermal cycler (Bio-Rad), and libraries generated according to the manufacturer’s protocol with no modifications (14 cycles used for cDNA amplification). For single cell libraries, Samples were sequenced on an Illumina HiSeq 4000 as 2 × 150 paired-end reads, one full lane per pool (before analysis, gene expression data were trimmed to 26 bp, read 1; 8 bp, i7 index; and 98 bp, read 2) or run on Illumina NovaSeq6000 with the same parameters (PE150, gene expression libraries trimmed to 28:8:0:98). Gene expression raw sequencing data were processed using CellRanger software v.3.0, and the VDJ TCR alpha and beta chains were processed using CellRanger VDJ v.3.1.0, both following the CellRanger pipeline. Sequencing reads were aligned to the mouse reference genome mm10 (Ensembl 93) provided by CellRanger.

### Bulk TCR β analysis

Total RNA was isolated with RNeasy Micro Kit (Qiagen) according to the manufacturer’s protocol and increasing RNA elution time to 10 minutes. Libraries were prepared using SMARTer Mouse TCR α/β Profiling kit (Takara Bio USA) following recommendations for 10-100ng of purified total RNA input and using primers for both α and β TCR chains. Final clean-up was performed with SPRIselect reagent (Beckman Coulter). Pooled libraries were sequenced with Illumina NextSeq 2000 P2 600 cycles kit 300PE with the 10% PhiX. Sequencing data were analyzed and clonal populations were identified using MiXCR v4.4.2 ((45) with recommended settings for SMARTer Mouse TCR α/β Profiling kit (analyze takara-mouse-rna-tcr-smarter). Cells were grouped by their TCR β sequences, and the top 200 largest EYFP+ clonal populations (by frequency in the tumor) from each mouse were used for subsequent analysis. Sequences matching public repertoires were removed (Immune Epitope Database).

### Adoptive Transfer

To determine the duration of tamoxifen activity, splenocytes were stimulated with anti-CD3 (2ug/ml) and anti-CD28 (10ug/ml) for 24 hours and intravenously transferred (3x10^6 cells/ mouse) into CD45.1 RAG2KO^-/-^ mice. In the B16-OVA experiment, CD44+CD8+ T cells (1 × 10^6/ mouse) from OT-I x AgRSR-LSL-EYFP mice were intravenously transferred into B16-OVA bearing mice, 10 days after tumor inoculation following published protocols (8).

### Processing of Antigen-signaled T cell Clonal Populations

From the scRNA/TCR-seq data, cells expressing identical TCR alpha and beta (V(D)J) nucleotide sequences were defined as antigen-signaled T cell clonal populations. Antigen-signaled T cell clonal populations were classified as antigen-signaled CD8+ or CD4+ T cell clonal populations if more than 60% of cells or less than 60% of cells in the population expressed *Cd8a* respectively. Antigen-signaled Treg clonal populations were computationally defined as antigen-signaled CD4+ T cell clonal populations in which more than 10% of cells expressed *Foxp3*. Downstream analysis was restricted to cells from the spleen and tumor, clonal populations of sizes greater than 15 with cells in the tumor, and in the case of the intratumoral dataset, clonal populations from mice that had no evidence of tamoxifen leakage. Clonal populations size (frequency) was calculated as the number of cells in the spleen and tumor of each clonal population as a fraction of the total captured T cells in the tissue compartment.

### Gene set Scores

The gene sets for cell cycle status were taken from Tirosh et al., 2016 (29) and the CellCycleScoring module was used to assign cells either a G1 or G2M/S phase (proliferating) status in Seurat. Gene sets from Li et al., (2018) (28), Bending et al., (2018) (34) and Yost et. al (2019) (46) (based on Im et al., (2017) (38)) were used to score cells for exhaustion, effector Treg differentiation and Tcf7 self-renewal capacity respectively. Additional gene sets from Lucca et al., (2021) (30), Wherry et al., (2007) (32) and Beltra et al., (2020) (47) (based on Bengsch et al., (2018) (31)) were used to corroborate results from the exhaustion score analysis. Mouse ortholog genes (48) (based on Ensembl Biomart version 87) were used when gene sets were derived from human data. Gene set scores were calculated by normalizing and taking the log of the raw gene counts obtained from the CellRanger output and applying the score_genes function from Scanpy with the required gene set, as in Tirosh et al., 2016 (29). Scores were produced for each cell, and these were then averaged across cell members of a clonal population (or other subsets of cells as indicated in the text) to calculate scores for each clonal population.

### Human Data

Data from Gueguen et al. 2021 (33) was downloaded from GSE162498 and reprocessed using the same analysis pipeline.

### Integrating the scRNA/TCR-seq data for clustering and pseudotime analysis

To prevent the variable TCR genes from contributing to downstream analysis, genes containing TRAV and TRBV in their gene name were removed from the gene-expression matrix. The resulting count matrices and V(D)J sequences were further processed using scRepertoire (49), Seurat (50) and Scanpy (51). In brief, the samples were demultiplexed with the aid of the HTODemux function, matched to their V(D)J sequences with the combineTCR and combineExpression functions, filtered (to cells that had full TCR alpha and beta (V(D)J) nucleotide sequences, feature counts in the 200-5000 range, and less than 10 percent mitochondrial counts), pre-processed by the SCTransform function, and integrated with the FindIntegrationAnchors and IntegrateData functions (52). Relevant cells were subset from the main dataset, and new Principal Component Analysis (PCA) (number of dimensions = 50), and Uniform Manifold Approximation and Projection (UMAP) coordinates were generated for Louvain clustering (53, 54) (resolution=1.5), and Monocle 3 (55) pseudotime analysis.

### TrajClust Algorithm

TrajClust was evaluated on simulated datasets to demonstrate that it could reveal shared differentiation patterns that would have otherwise been hidden (Further details in Supplmentary Methods, Development and Testing of TrajClust). TrajClust was applied to clonal populations from the days 8 and 18 intraperitoneal tamoxifen datasets, restricted to those of size 75 or greater. One clonal population with a distinct differentiation state was considered an outlier and removed from the dataset prior to analysis. The hierarchical clustering results were flattened to discrete clusters by choosing the cluster size that maximized the silhouette score of the resulting clusters. This resulted in 4 clusters denoted clonal differentiation patterns.

### GLIPH2 Analysis

GLIPH2 (56) using CDR3a, CDR3b, TRBV and TRAJ sequences was applied to all clonal populations of size greater than 15 from the days 8 and 18 intraperitoneal tamoxifen datasets to uncover common reactivity groups. Some clonal populations were assigned to multiple reactivity groups. This was represented in Fig. 6E by equally dividing a clonal population’s allocation in the pie chart to their respective reactivity groups.

### Software Versions

Data was analyzed using R version 4.0.3 and R packages (Seurat 4.0.4, SeuratData 0.2.1, SeuratDisk 0.0.0, scRepertoire 1.0.0, splatter 1.14.1 and monocle3 0.2.3), Python version 3.8.6 and Python packages (jupyterlab 2.2.9, numpy 1.19.4, pandas 1.1.5, scipy 1.6.0, scanpy 1.6.0, anndata 0.7.5, rpy2 3.3.6, anndata2ri 1.0.5.dev2+ea266ab, skmisc 0.1.3, sktime 0.5.2, scikit-learn 0.24.1 and tqdm 4.54.1). Figures were produced with seaborn 0.11.0, matplotlib 3.5.1 in Python, Prism 10, Affinity Publisher 1.10.8, and using illustrations from Irasutoya (https://www.irasutoya.com/).

### Statistical Analysis

Statistical analyses on the FACS data were performed using two-tailed Student’s t-tests unless otherwise indicated. Differentially expressed genes were recorded for genes with p<0.01 and log2F>0.5. Paired comparisons were assessed for statistical significance using Kruskal-Wallis with Scipy.

## Supplementary Material

Supplementary Materials

Supplementary Data Sheet 1

Supplementary Data Sheet 2

## Data Availability

Sequence data that support the findings of this study have been deposited in the Gene Expression Omnibus database (GEO: in preparation). Code for next-generation sequencing analysis and TrajClust is deposited on Zenodo (https://doi.org/10.5281/zenodo.11057548) (57) and Github (https://github.com/MunetomoT/TrajClust) respectively. Raw data is included in Supplementary Data File S2. Additional information and materials will be made available upon request.

## One-Sentence Summary

An antigen signaling fate-mapping mouse reveals intratumoral antigen signals trap CD8+ T cell clonal populations in tumors.
