# Supplementary material for: Intratumoral antigen signaling traps CD8+ T cells to confine exhaustion to the tumor site
Source: Sci Immunol. Author manuscript; Available in PMC 2024 Jul 16. (PMC7616235; doi:10.1126/sciimmunol.ade2094)
Supplement: Supplementary Materials [file EMS197228-supplement-Supplementary_Materials.pdf]

Science Immunology
AAAS

Supplementary Materials for

Intratumoral antigen signaling traps CD8$^+$ T cells to confine exhaustion to the tumor site

Munetomo Takahashi *et al.*

Corresponding author: James E. D. Thaventhiran, jedt2@cam.ac.uk

Sci. Immunol. **9**, eade2094 (2024)
DOI: 10.1126/sciimmunol.ade2094

The PDF file includes:

Supplementary Text
Figs. S1 to S10
References (*58–66*)

Other Supplementary Material for this manuscript includes the following:

Data files S1 and S2
MDAR Reproducibility Checklist

Supplementary Text

Development and Testing of TrajClust

Overview

In recent years, technologies capturing data at single cell resolution combining a cell's transcriptomic data with clonality have allowed the differentiation states of clonal populations to be explored in vivo (*58, 59*). However, current methods to cluster clonal differentiation patterns assume a homogenous differentiation state for clonal populations, and consequently do not compare the clonal populations' whole differentiation trajectory. We developed an algorithm, TrajClust to cluster clonal differentiations based on their differentiation trajectory. We found that TrajClust could uncover groups of clonal differentiations that were not identifiable by standard approaches leveraging UMAP (*60*) representations in both synthetic and real-world data. TrajClust therefore demonstrates the limitations of inferring clonal characteristics from low-dimensional representations and provides a novel approach to uncover groups of clonal differentiation patterns in a variety of settings.

Background

The TrajClust algorithm aims to infer groups of similar clonal differentiation patterns (Fig. S8A), from single cell RNA datasets using information about each cell's clonal population (matching TCR alpha and beta chains in the case of T cells).
Standard approaches analyze similarities between clonal populations by their differentiation state distributions. Cells are first clustered after dimensionality reduction, and the clonal populations are then grouped by either the cluster most of their cells belong to or by the distribution of their cells in each cluster (*39-41*). These approaches have limitations. Firstly, they focus on the differentiation states occupied by a clonal population rather than their differentiation trajectory. Consequently, clustering results may be biased by the proliferative capacity of each differentiation state. Secondly, these standard approaches assume that key information about clonal populations similarities is always retained after dimension reduction. A clonal population's similarity with another clonal populationcould be lost if it is assumed that cells in the same differentiation state are homogeneous. The heterogeneity within differentiation states could be better utilized to infer similarities between clonal differentiation trajectories.

TrajClust Algorithm

TrajClust first infers the pseudotime of cells using Monocle 3 (*56*). This introduces a transcriptional ordering of cells, providing an additional axis by which to compare clonal populations. The genes are normalized per cell, log(x+1) transformed, optionally imputed using MAGIC (*61*) as implemented in Scanpy 1.6.0 (*52*) and the gene counts are then scaled in accordance with the current best practices (*62*). The gene expressions of cells within a clonal populations produces gene trajectories across pseudotime. These trajectories are smoothed by locally estimated scatterplot smoothing (LOESS) (*63*) as implemented in scikit-misc (*64*) and sampled at 0.5 (AU) pseudotime intervals. The distances between clonal populations (each with corresponding gene trajectories X, and Y) are then computed as the dynamic time warping (DTW) distances of their corresponding gene trajectories.

$$DTW(X,Y) = \sqrt{\sum_{(i,j)\in\pi} \|X_i - Y_j\|^2}$$

These distances are calculated with the sktime package (*65*). Finally, hierarchical clustering on the resultant distance matrix with ward linkage uncovers clusters of clonal populations.

Evaluation

TrajClust was evaluated on both synthetic and real datasets and shown to outperform standard clustering approaches in both cases (Fig. S8B).

Synthetic Dataset

Synthetic datasets were created utilizing the Splatter framework (*66*). Each dataset comprised of a count matrix of 1000 genes over 5000 cells (50 clonal populations of size 100 categorized into 5 clonal differentiation patterns based on gene trajectory adjustments) that differentiate across 4 cell states. For each clonal differentiation pattern, a selected number of common genes were adjusted. The gene trajectories were adjusted by first fitting the trajectories onto a basis function:

$$\text{uturn basis } (u\text{-basis}) = \pm\frac{L-H}{P}(x-P)^2 + \{H \text{ or } L\},$$

$$\text{linear basis } (l\text{-basis}) = \pm\frac{dy}{dt}(t-t_0) + L,$$

where L is the lowest gene count, H is the highest gene count, P is the location of inflex (equated to maximum pseudotime value for the evaluation), dy/dt is the range of gene counts over the range of pseudotime values and t_0 is the minimum pseudotime value. New gene expression counts were computed as the average between the basis function prediction count for the given pseudotime value and the original count.

Implementation of ‘Standard Approaches’

The performance of TrajClust was compared to clustering results of clonal populations obtained from standard approaches utilized in the literature (*39-41*). The datasets were normalized per cell, log(x+1) transformed, gene counts scaled and a neighborhood graph of observations was computed using standard parameters from Scanpy (*52*). The cells were then clustered using the Louvain cluster algorithm (*54, 55*) with a resolution parameter set to 50 (denoted Standard Approach). This parameter was varied in increments of 0.5 between 0 and 60 to find the resolution that maximized the clustering score (denoted Optimal Standard Approach). The former reflected the practical performance of the Standard Approach, whereas the latter reflected the theoretical maximum performance that could be achieved if the optimum resolution for clustering was known beforehand. In both cases, the cluster distribution matrix of clonal populations was hierarchically clustered with ward linkage to uncover different clonal differentiation patterns. TrajClust was implemented without MAGIC imputation.

First, we undertook an exploratory comparison of TrajClust with the Standard Approach (Fig. S8C) on synthetic datasets where the number of genes, and the type of adjustments were varied. The clusters produced by each algorithm were evaluated based on normalized mutual information (NMI) and adjusted rand index (ARI). Whilst both approaches could

discriminate different clonal differentiation patterns when many genes were adjusted, TrajClust was able to significantly outperform the Standard Approach when differences between clonal differentiation patterns were slight (20 genes, with U-basis/L-basis ratio set to 0.80) (Fig. S8D). This was still the case when the parameter chosen for the resolution in the Standard Approach was optimized (Fig. S8E).
We tested the robustness of TrajClust to outliers and noise. We created new synthetic datasets containing outlier clonal populations (additional clonal differentiation pattern(s) with a single clonal population) and evaluated the performance of the approaches (Fig. S8F). To simulate noise between the clonal differentiation patterns, we altered the original synthetic datasets so that the adjusted genes of clonal populations overlapped with one another. We added Gaussian noise to the overlapping gene trajectories and evaluated the performance of the approaches (Fig. S8G). In both cases, TrajClust outperformed the Standard Approach.

Real Dataset

We applied TrajClust to the days 8 and 18 intraperitoneal dataset as described in the main text. To minimize the effect of under sampling and missed capture of a clonal population's cells on the calculated differentiation trajectory we considered large clonal populations of size greater than 75 (42 clonal populations) and utilized MAGIC imputation. TrajClust uncovered four groups of clonal differentiation patterns which the Standard Approach did not (Fig. S8H).

Fig. S1

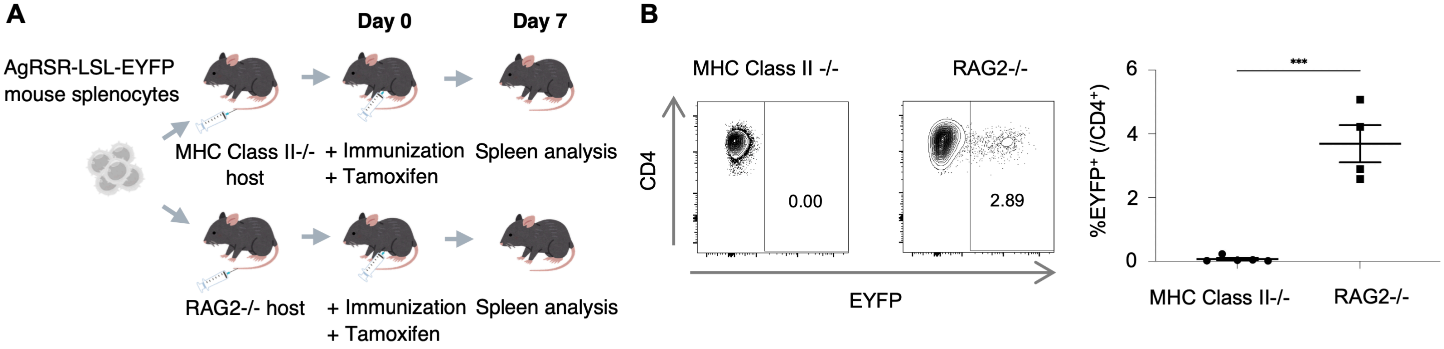

Fig. S1 The AgRSR mouse fate-maps antigen-signaled CD4+ T cells, related to Fig. 1.
(**A**) AgRSR-LSL-EYFP splenocytes were adoptively transferred to CD4+ T cell deficient MHC Class II$^{-/-}$ and RAG2$^{-/-}$ strains, challenged with OVA (with anti-CD40 and poly I:C) and tamoxifen treated. (**B**) Representative flow cytometry plots of EYFP and CD4 expression in splenocytes at day 7 post immunization (Left), and summary plot of all experiments (Right). Dots represent mice (**B**). Mean ± SEM as shown (**B**). Statistical testing via unpaired two-tailed students t-test (***, p <0.001).

Fig. S2

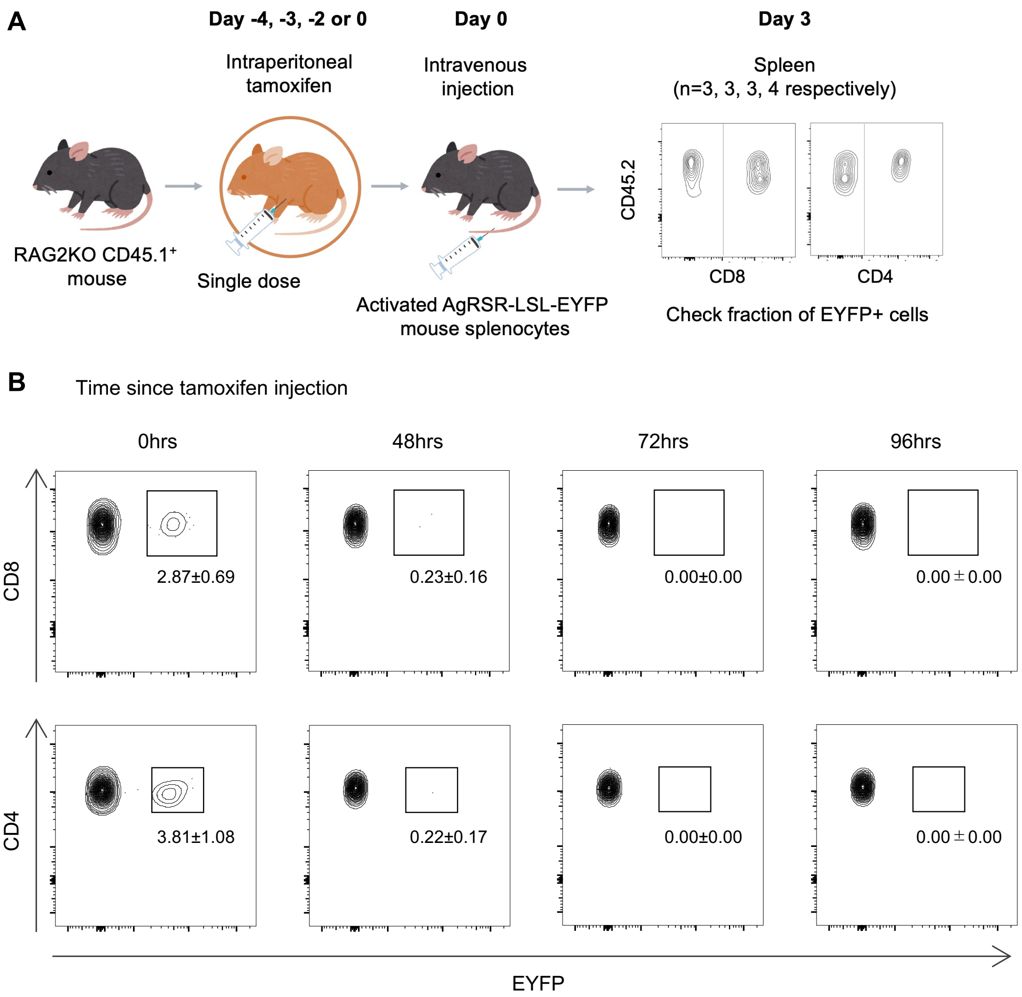

Fig. S2. Evaluating the duration of tamoxifen signaling *in vivo*.
(**A**) Activated CD45.2 AgRSR-LSL-EYFP splenocytes were intravenously injected into CD45.1+ RAG2-/- that had received intraperitoneal tamoxifen injections 0, 48, 72 or 96 hours (hrs) before. (**B**) Representative flow plots, and %EYFP of CD8+ (Top) and CD4+ (Bottom) T cells from CD45.2+ cells in the spleen on different days after intraperitoneal tamoxifen injection. Mean and SEM shown. (n=3, 3, 3, 4 for each day respectively).

Fig. S3

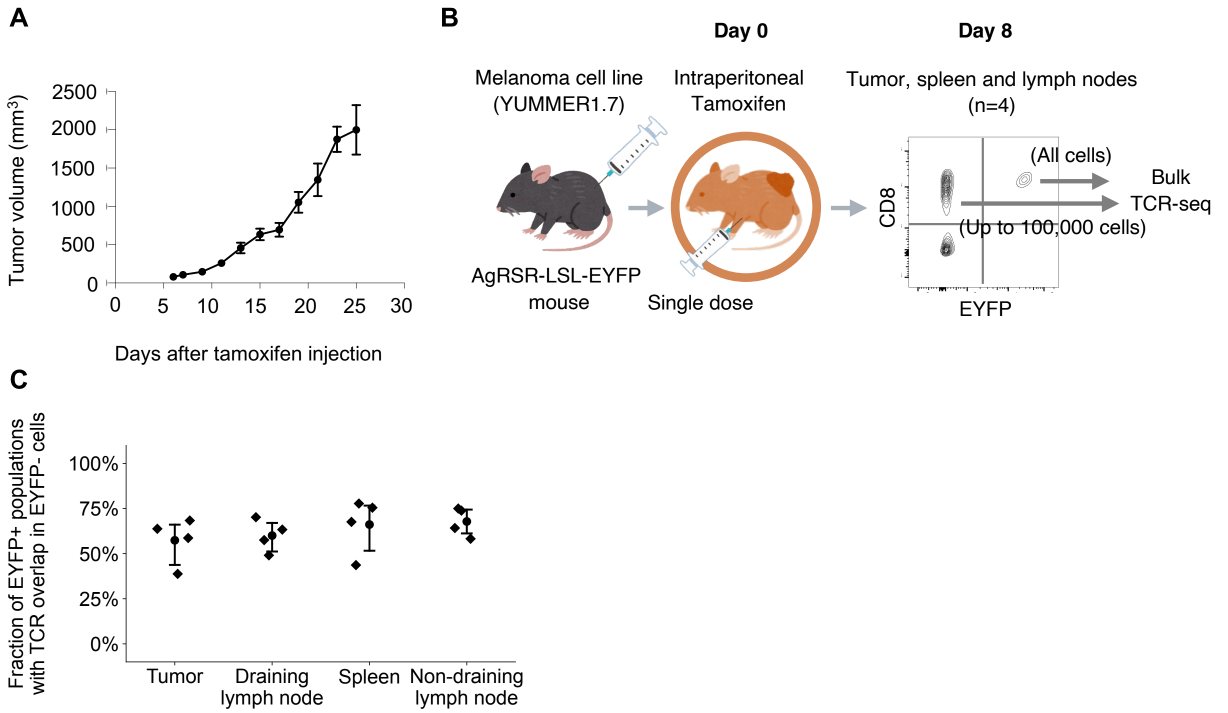

Fig. S3. Tracking antigen-signaled CD8+ T cell responses in the YUMMER1.7 model, related to Fig. 2.
(**A**) Change in YUMMER1.7 tumor volume over time after implantation in AgRSR-LSL-EYFP mice receiving tamoxifen at day 7. Data are representative of 5 independent experiments (n=3-11 per condition). (**B**) 8 days after intraperitoneal tamoxifen injection, EYFP+ and EYFP- CD8+ T cells were sorted from the tumor, draining, spleen (pre-enriched for T cells) and non-draining lymph node of AgRSR-LSL-EYFP mice and subject to bulk TCR-seq analysis (n=4). The top 200 largest EYFP+ CD8+ T cell clonal populations in the tumor of each mouse was analyzed. (**C**) Fraction of EYFP+ clonal populations that had TCR overlap with EYFP- cells in the indicated tissues. Dots represent mice (**C**).

Fig. S4

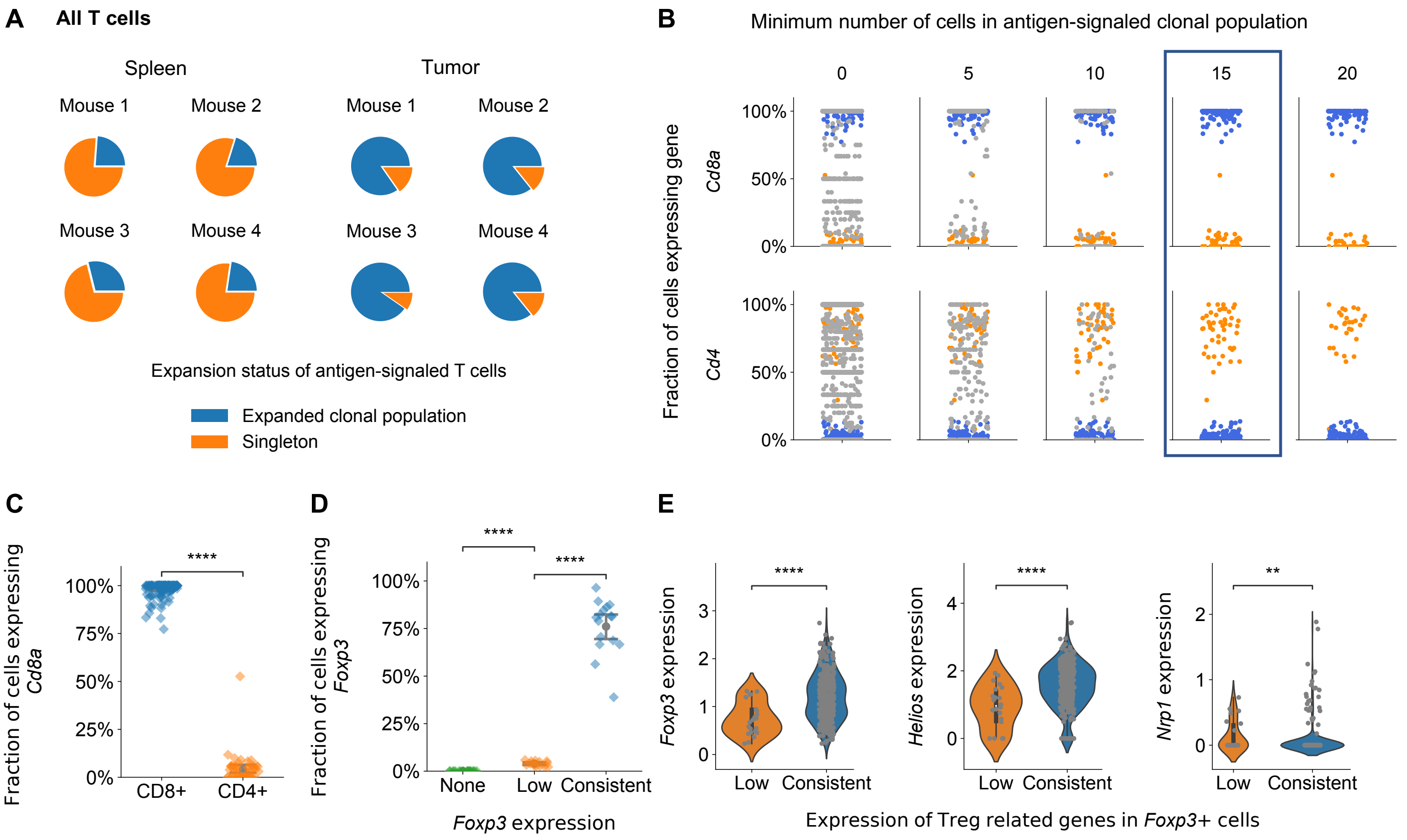

Fig. S4. Exploring the characteristics of antigen-signaled T cell clonal populations 8 days after intraperitoneal tamoxifen administration, related to Fig. 3.

(**A**) Proportion of T cells obtained in scRNA-seq analysis that were identified as members of expanded clonal populations or singleton (non-expanded) for data obtained in Fig. 3A. (**B**) Fraction of cells expressing *Cd8a* (Top) and *Cd4* (Bottom) in each antigen-signaled T cell clonal population. Minimum size (number of cells) of clonal population varied as shown. Antigen-signaled clonal populations are colored blue (CD8+), orange (CD4+) or grey (undefined) based on definitions used in Fig. S3C and subsequent analysis. (**C**) Fraction of cells expressing *Cd8a* for the largest antigen-signaled clonal populations. (**D**) Grouping antigen-signaled CD4+ T cell clonal populations based on fraction of cells expressing *Foxp3*. Populations are categorized as not expressing *Foxp3* (0% of cells have detectable *Foxp3* expression), low *Foxp3* expression (less than 10% of cells have detectable *Foxp3* expression) and consistent *Foxp3* expression (more than 10% of cells have detectable *Foxp3* expression). (**E**) Violin plots comparing the expression of Treg-related genes in *Foxp3* expressing cells from low and consistent *Foxp3* expressing antigen-signaled clonal populations. Dots represent cells (**E**) and antigen-signaled clonal populations (**B-D**). Statistical testing via Kruskal-Wallis test (****, p <0.0001; **, p <0.01).

Fig. S5

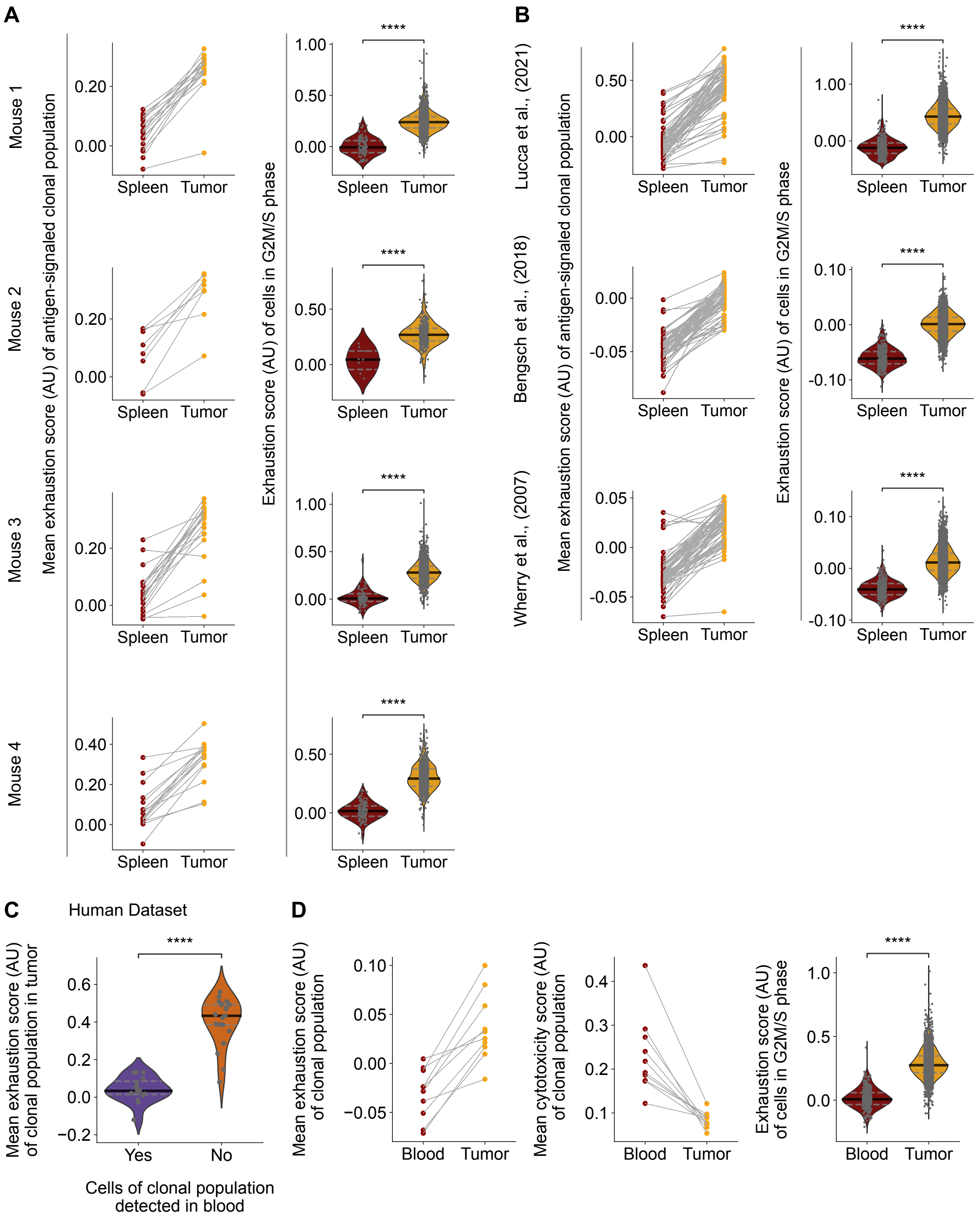

Fig. S5. Exploring the differentiation states of antigen-signaled CD8+ T cell clonal populations 8 days after intraperitoneal tamoxifen administration related to Fig. 3 and S4. (**A-B**) Reproducing results of Fig. 3C and E for individual mice (**A**) and by using different exhaustion gene sets (**B**) as indicated. (**C-D**) CD8+ T cell clonal populations (grouped by TCR)

from patients with lung cancer. (**C**) Comparing the mean exhaustion score of cells in the tumor of clonal populations that had clonally related cells detected in blood with those that did not. (**D**) Clonal populations with at least two cell members in both the tumor and blood were assessed for mean exhaustion (Left) and cytotoxicity (Middle) gene set expression score in each tissue. Scores from the same clonal population are linked by a line. Violin plot comparing the exhaustion gene set scores of individual cells in G2M/ S phase across the blood and tumor (Right). Dots represent cells (**A-B**, **D**), clonal populations (**C-D**) and antigen-signaled clonal populations (**A-B**). Statistical testing via Kruskal-Wallis test (****, $p < 0.0001$). (AU) arbitrary units.

Fig. S6

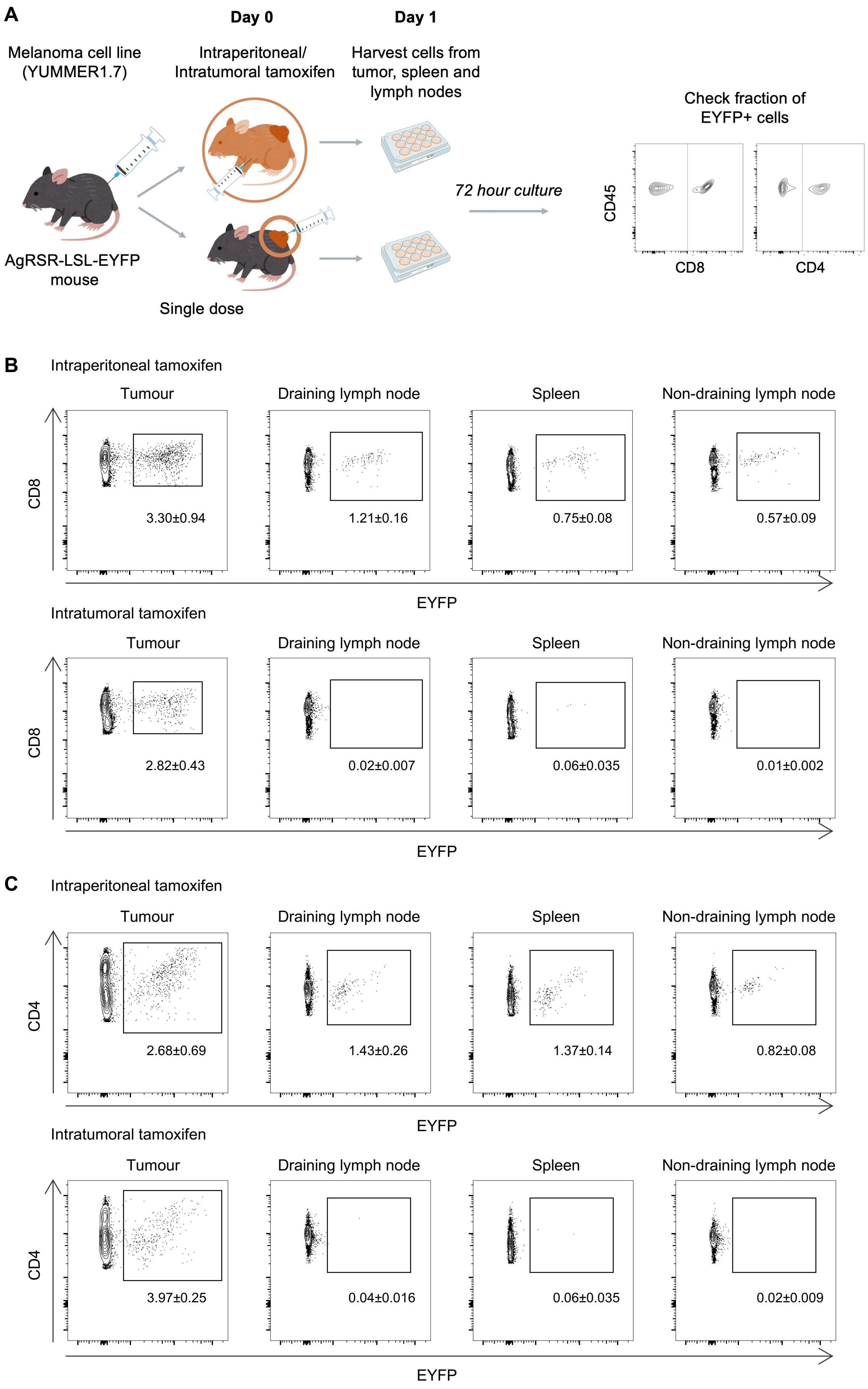
A
Day 0
Day 1
Melanoma cell line (YUMMER1.7)
Intraperitoneal/ Intratumoral tamoxifen
Harvest cells from tumor, spleen and lymph nodes
Check fraction of EYFP+ cells
72 hour culture
CD45
CD8
CD4
AgRSR-LSL-EYFP mouse
Single dose
B
Intraperitoneal tamoxifen
Tumour
Draining lymph node
Spleen
Non-draining lymph node
CD8
3.30±0.94
1.21±0.16
0.75±0.08
0.57±0.09
EYFP
Intratumoral tamoxifen
Tumour
Draining lymph node
Spleen
Non-draining lymph node
CD8
2.82±0.43
0.02±0.007
0.06±0.035
0.01±0.002
EYFP
C
Intraperitoneal tamoxifen
Tumour
Draining lymph node
Spleen
Non-draining lymph node
CD4
2.68±0.69
1.43±0.26
1.37±0.14
0.82±0.08
EYFP
Intratumoral tamoxifen
Tumour
Draining lymph node
Spleen
Non-draining lymph node
CD4
3.97±0.25
0.04±0.016
0.06±0.035
0.02±0.009
EYFP

Fig. S6. Quantifying enrichment for marking intratumorally antigen-signaled T cells with intratumoral tamoxifen injection, related to Fig. 4 and 5.
(**A**) YUMMER1.7 bearing mice were injected with intraperitoneal or intratumoral tamoxifen. Single cell suspensions were extracted from the tumor, spleen (pre-enriched for T cells), draining and non-draining lymph nodes and cultured for 72 hours in IL-2 and IL-7. (**B-C**) Representative flow plots, and %EYFP of CD8+ (**B**) and CD4+ (**C**) T cells from different tissues after intraperitoneal (Top row of each figure) and intratumoral (Bottom row of each figure) injection. Mean and SEM shown (n=5 and 2 respectively).

Fig. S7

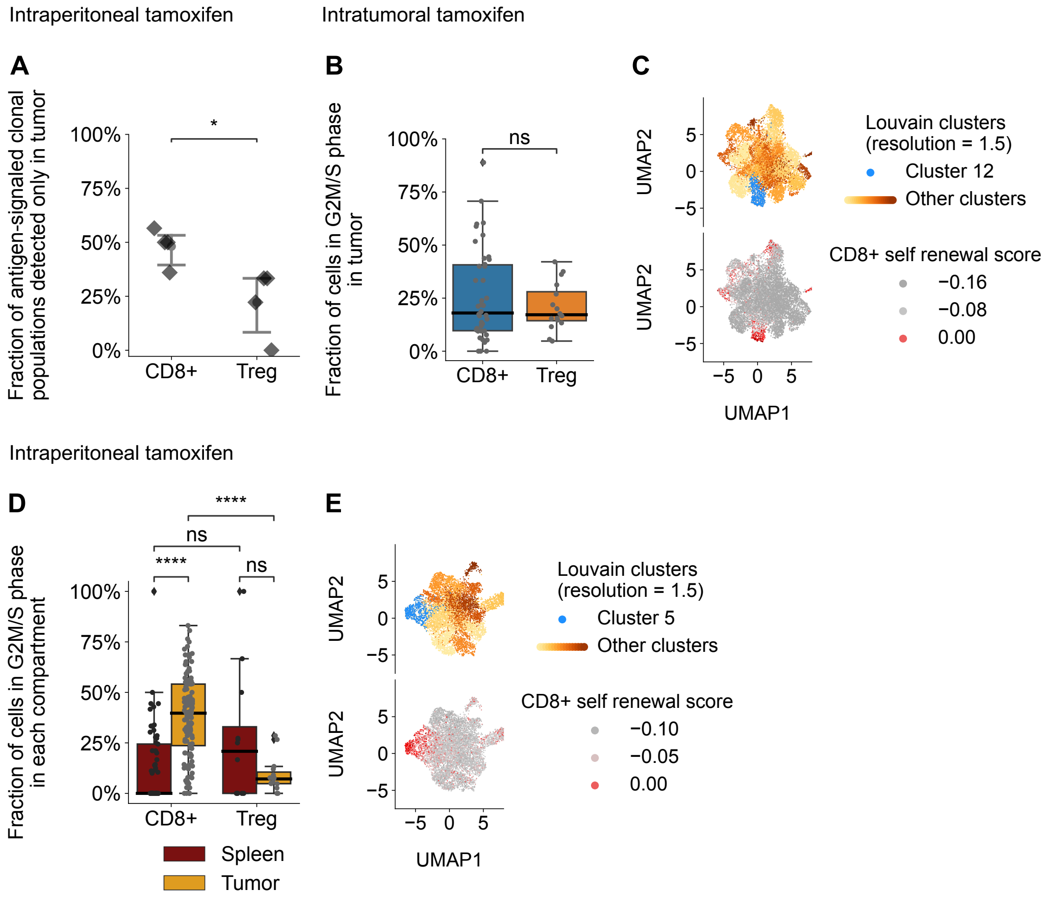

Fig. S7. Characterizing intratumorally antigen-signaled T cell clonal populations, related to Fig. 5.
(**A**) Fraction of antigen-signaled CD8+ T cell and Treg clonal populations for which no cells with the same TCR were identified in EYFP+ cells of the spleen from scRNA/TCR-seq dataset after intraperitoneal tamoxifen labelling. (**B-E**) Characteristics of antigen-signaled CD8+ T cell clonal populations from intratumoral (**B-C**) and intraperitoneal (**D-E**) tamoxifen injection. (**B**) Boxplots showing the fraction of cells in the tumor in G2M/ S phase. (**C**) UMAPs of tumoral CD8+ T cells colored by Louvain clusters (Top) with highlighting of cluster highly expressing a self-renewal gene set (Bottom). (**D**) Boxplot showing the fraction of cells in G2M or S phase for antigen-signaled CD8+ T cell and Treg clonal populations within the indicated tissue compartment. (**E**) UMAPs of tumoral CD8+ T cells colored by Louvain clusters (Top) with highlighting of clusters highly expressing a self-renewal gene set (Bottom). Dots represent cells (**C, E**), antigen-signaled clonal populations (**B, D**) and mice (**A**). Statistical testing via paired two-tailed students t-test and Kruskal-Wallis test (****, p <0.0001; *, p<0.05; ns, p>0.05).

Fig. S8

A

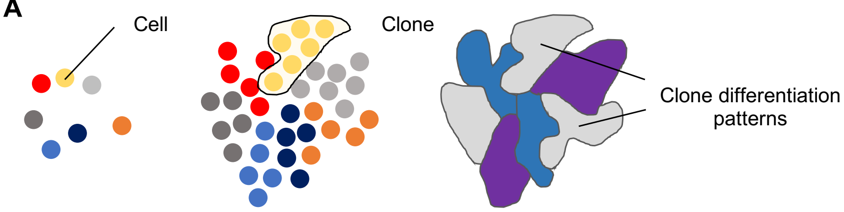

D E F G

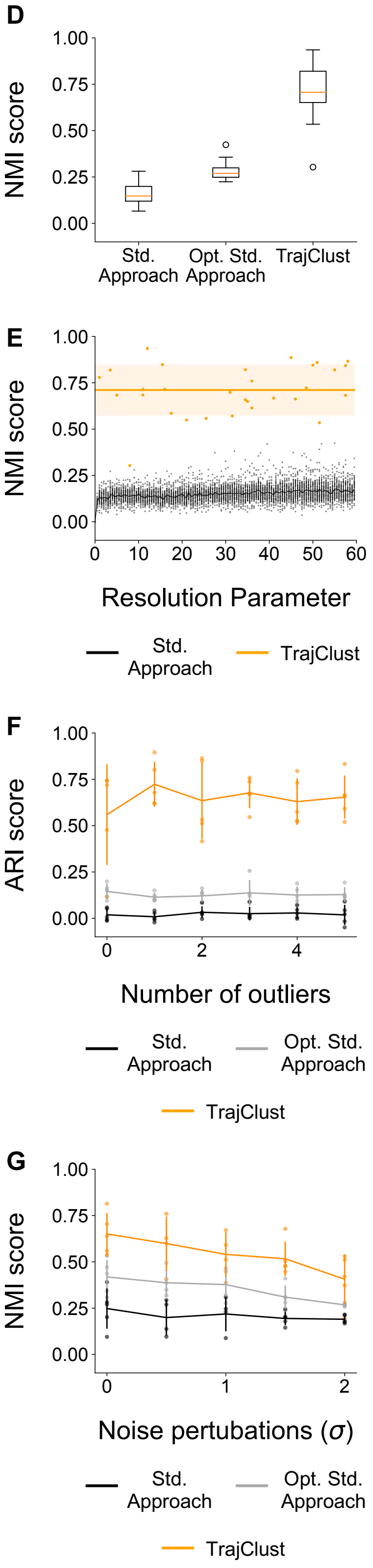

B

	NMI score			
	**Std. Appr.**	**Opt. Std. Appr.**	**TrajClust**	***p*-value**
**Performance (synthetic)**				
20 genes perturbed	0.162	0.279	**0.711**	**3.29e-18**
**Robustness (synthetic)**				
Outliers (3 to 5)	0.310	0.420	**0.798**	**6.10e-5**
Noise (1.0σ to 2.0σ)	0.200	0.313	**0.484**	**3.66e-4**
**Performance (real)**				
Biological interpretation	None	None	**4 differentiation patterns**	
Score	0.434	0.436	**0.729**	

C

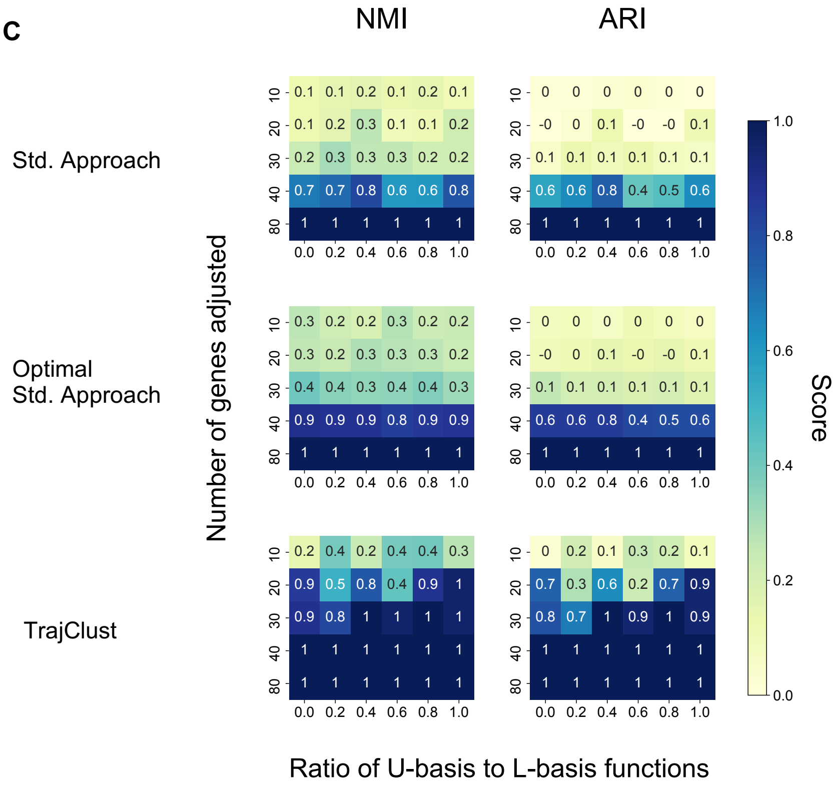

H

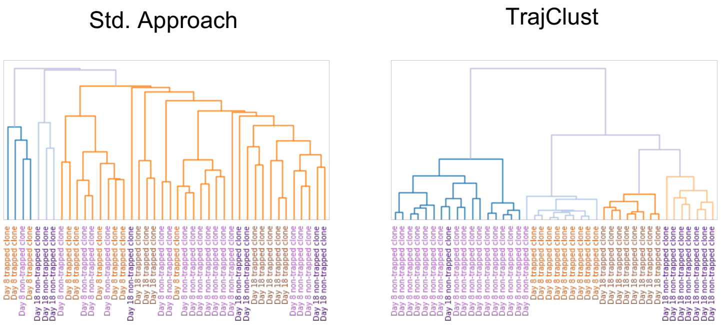

Fig. S8. The development, evaluation, and performance of TrajClust.
(**A**) Diagram illustrating the clonal differentiation patterns that the algorithm aims to uncover. (**B**) Overview table of normalized mutual information (NMI) scores of each approach evaluated over synthetic and real datasets. Std. Appr. refers to the 'Standard Approach' with the resolution parameter set to 50, Opt. Std. Appr. refers to a theoretical optimum of the 'Standard Approach' wherein the resolution parameter is optimized to produce the best NMI score. p-value calculated between the NMI scores of the Opt. Std. Approach and the TrajClust algorithm on the relevant datasets using unequal variances t-test for Performance comparisons (n=30 per comparison) and Wilcoxon signed-rank test for Robustness comparisons (n=13-15 per comparison). (**C**) NMI and adjusted rand index (ARI) scores (rounded to 1 significant figure) for different synthetic datasets. The number of genes adjusted, and the type of adjustment was altered to produce 30 unique synthetic datasets. (**D**) Statistical comparison by unequal variances t-testing of the approaches on 30 synthetic datasets where 20 genes were adjusted with a u-basis to l-basis ratio set to 0.80. (**E**) The same dataset as used in (**D**) plotted over alterations of the resolution parameter. (**F**) ARI score of approaches after outliers are introduced to one of the datasets in (**D**) with mean and standard deviations as shown (n=5 per condition). ARI score used to account for effect of chance. (**G**) NMI score of approaches after Gaussian noise is introduced to new synthetic dataset with mean and standard deviations as shown (n=4-5 per condition). All gene adjustments overlapped across the different clonal differentiation patterns in the new synthetic dataset. (**H**) Dendrogram result of Standard Approach (Left) and TrajClust algorithm (Right) applied to the mice dataset. Dendrogram colored by four largest clusters. Clonal populations colored by their topographic and time elapsed since antigen signaling characteristic.

Fig. S9

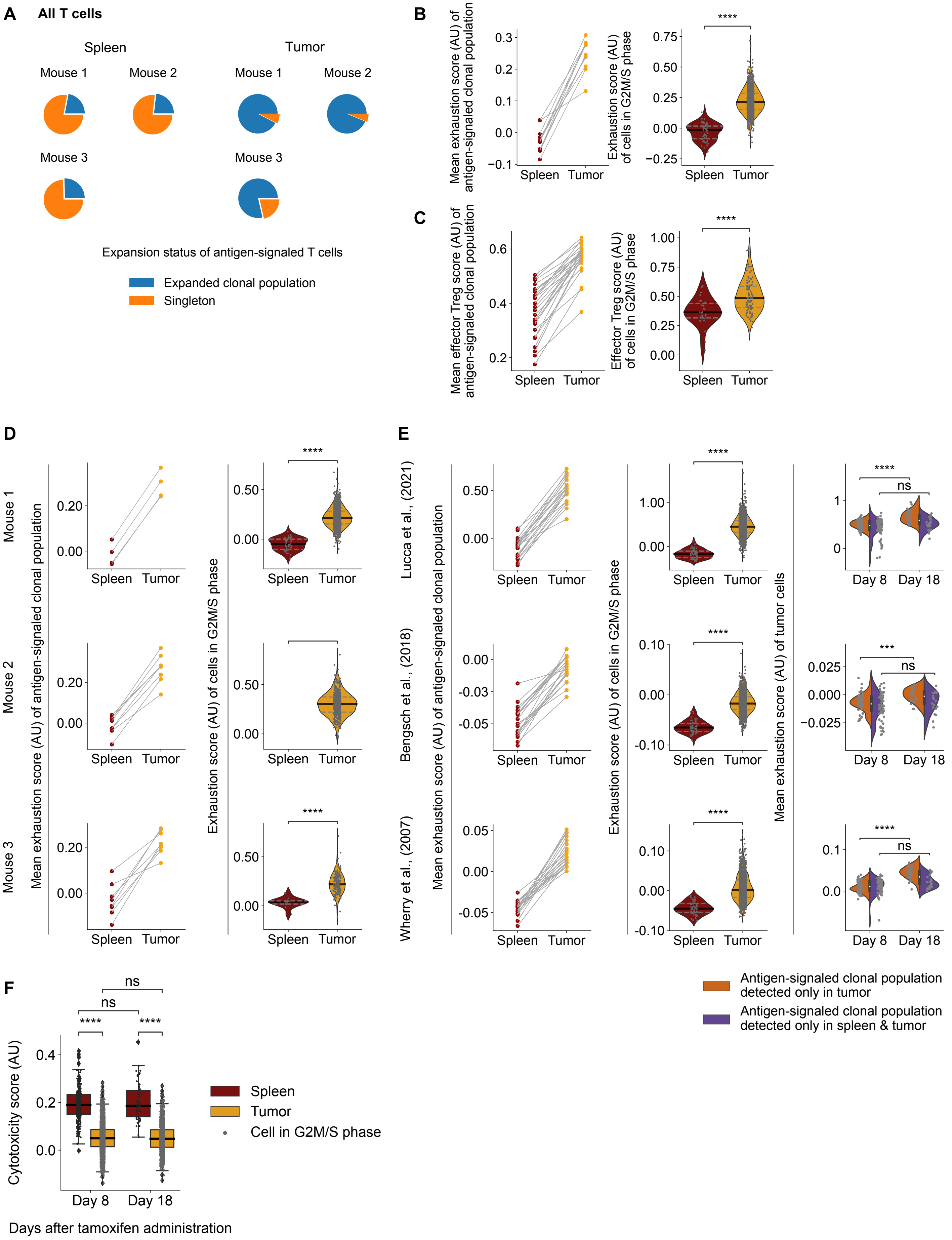
A
All T cells
Spleen
Tumor
Mouse 1
Mouse 2
Mouse 3
Expansion status of antigen-signaled T cells
Expanded clonal population
Singleton
B
Mean exhaustion score (AU) of antigen-signaled clonal population
Exhaustion score (AU) of cells in G2M/S phase
Spleen
Tumor
****
C
Mean effector Treg score (AU) of antigen-signaled clonal population
Effector Treg score (AU) of cells in G2M/S phase
D
Mean exhaustion score (AU) of antigen-signaled clonal population
Exhaustion score (AU) of cells in G2M/S phase
E
Lucca et al., (2021)
Bengsch et al., (2018)
Wherry et al., (2007)
Mean exhaustion score (AU) of tumor cells
Day 8
Day 18
***
ns
Antigen-signaled clonal population detected only in tumor
Antigen-signaled clonal population detected only in spleen & tumor
F
Cytotoxicity score (AU)
Days after tamoxifen administration
Spleen
Tumor
Cell in G2M/S phase

Fig. S9. Exploring the characteristics of antigen-signaled T cell clonal populations 18 days after intraperitoneal tamoxifen administration, related to Fig. 7.
(**A**) Proportion of T cells obtained in scRNA-Seq analysis that were identified as members of expanded clonal populations or singleton (non-expanded) for data obtained in Fig. 6A. (**B-C**) Reproducing results of Fig. 3C, E, F and G for antigen-signaled CD8+ T cell (**B**) and Treg (**C**) clonal populations 18 days after intraperitoneal marking. (**D-E**) Reproducing results of (**B**) for individual mice (**D**) and by using different exhaustion gene sets (**E**). Mouse 2 had no cells in G2M/ S phase in the spleen. (**F**) Boxplot comparing the cytotoxicity scores of CD8+ T cells in G2M/ S phase from the spleen and tumor at days 8 and 18. Dots represent cells (**B-F**) and clonal populations (**B-E**). Statistical testing via Kruskal-Wallis test (****, $p < 0.0001$; ***, $p < 0.001$; ns, $p > 0.05$). (AU) arbitrary units.

Fig. S10

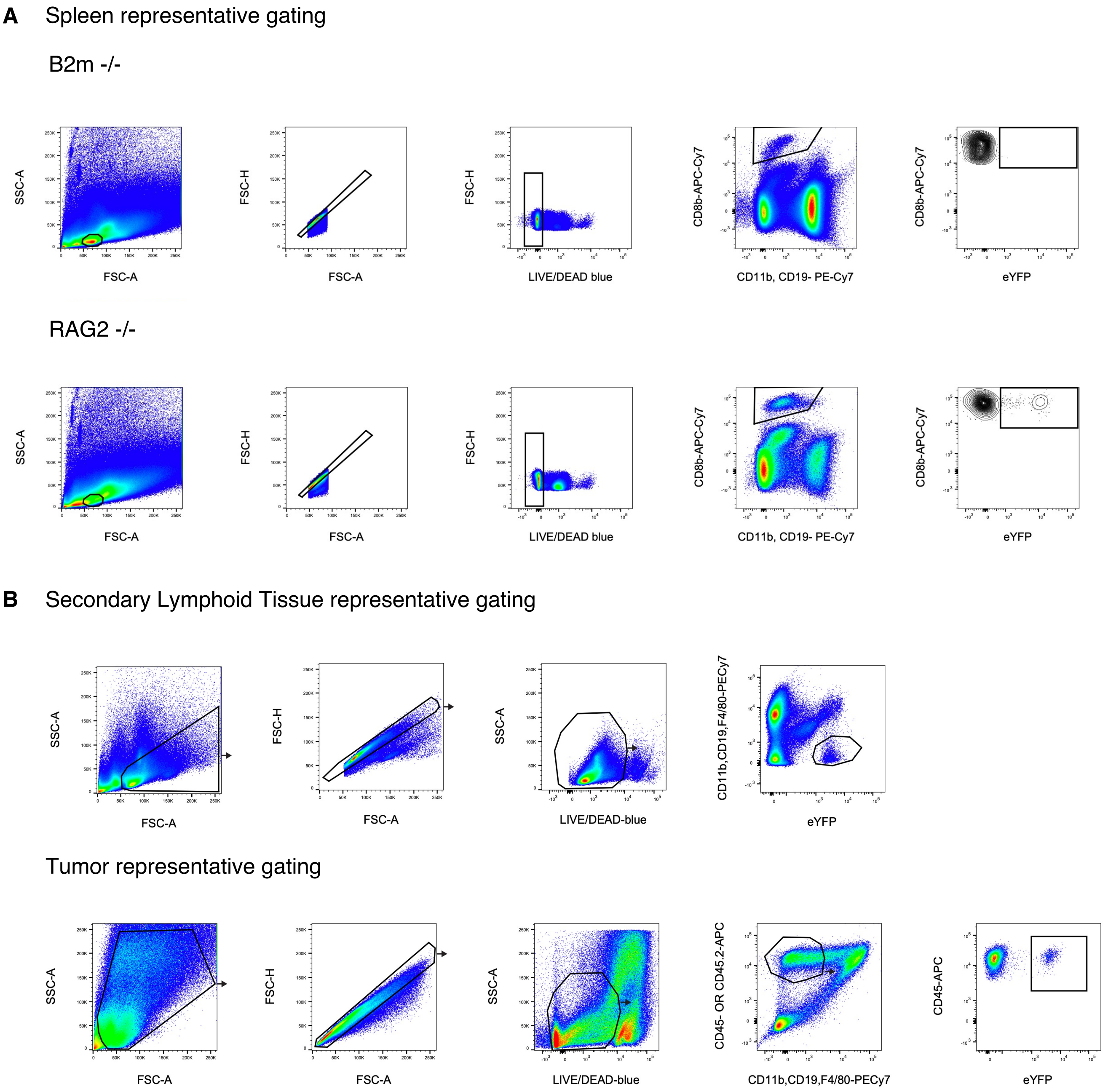

Fig. S10. Representative flow cytometry gating.
(**A**) Example gating for the analysis of EYFP+ T cells from the B2m-/- and RAG2-/- recipient mice. (**B**) Example gating for the analysis of EYFP+ T cells from secondary lymphoid tissue (Top) and tumor (Bottom).
